# Nanomaterials: small particles show huge possibilities for cancer immunotherapy

**DOI:** 10.1186/s12951-022-01692-3

**Published:** 2022-11-16

**Authors:** Ziyin Chen, Ziqi Yue, Kaiqi Yang, Shenglong Li

**Affiliations:** 1 Department of Urology, China‐Japan Friendship Hospital, Beijing, 100029 People’s Republic of China; 2 School of Basic Medicine, Harbin Medical University, Harbin, 150001 People’s Republic of China; 3 Department of Bone and Soft Tissue Tumor Surgery, Cancer Hospital of Dalian University of Technology, Cancer Hospital of China Medical University, Liaoning Cancer Hospital & Institute, No. 44 Xiaoheyan Road, Shenyang, 110042 Liaoning People’s Republic of China

**Keywords:** Nanomaterials, Immunotherapy, Tumor microenvironment, Tumor immunosuppressive microenvironment, Anti-tumor treatment

## Abstract

With the economy's globalization and the population's aging, cancer has become the leading cause of death in most countries. While imposing a considerable burden on society, the high morbidity and mortality rates have continuously prompted researchers to develop new oncology treatment options. Anti-tumor regimens have evolved from early single surgical treatment to combined (or not) chemoradiotherapy and then to the current stage of tumor immunotherapy. Tumor immunotherapy has undoubtedly pulled some patients back from the death. However, this strategy of activating or boosting the body's immune system hardly benefits most patients. It is limited by low bioavailability, low response rate and severe side effects. Thankfully, the rapid development of nanotechnology has broken through the bottleneck problem of anti-tumor immunotherapy. Multifunctional nanomaterials can not only kill tumors by combining anti-tumor drugs but also can be designed to enhance the body's immunity and thus achieve a multi-treatment effect. It is worth noting that the variety of nanomaterials, their modifiability, and the diversity of combinations allow them to shine in antitumor immunotherapy. In this paper, several nanobiotics commonly used in tumor immunotherapy at this stage are discussed, and they activate or enhance the body's immunity with their unique advantages. In conclusion, we reviewed recent advances in tumor immunotherapy based on nanomaterials, such as biological cell membrane modification, self-assembly, mesoporous, metal and hydrogels, to explore new directions and strategies for tumor immunotherapy.

## Introduction

Despite the trend of diversification and individualization of oncology treatment options, the mortality rate and the number of deaths from oncology have become substantial health and economic burden worldwide with the growing population and aging [1–4]. How to overcome the high mortality rate and poor prognosis of tumors is still an issue worth discussing today with continuous medical breakthroughs. Among the various strategies of tumor treatment, immunotherapy has a long history of clinical application in addition to traditional surgery and chemoradiotherapy regimens. Notably, an American physician William Coley, proposed and used inactivated bacteria to fight against tumors as early as the late twentieth century [5]. This anti-tumor immunotherapy based on Coley's toxin has made him known as the father of cancer immunotherapy [6, 7]. However, this pioneering tumor treatment option did not receive enough attention due to the limitations of science and technology. But with the flourishing of genetic engineering, synthetic technologies, and high-throughput sequencing, it has been confirmed that the immune system plays a decisive role in many aspects of tumor origin, progression, and drug resistance [8–11]. Moreover, the interaction between tumors and the immune system is not only about nutrient supply and signal transduction but also forms the tumor microenvironment (TME), which is the hotbed of tumor growth, invasion and metastasis. Even tumor cells train immune cells in this environment to achieve immune escape and suppression [12–14].

According to the critical role of the immune system in the tumor, the clinical application of immunotherapy as a representative of new generation treatment options is gradually expanding. To date, the FDA (Food and Drug Administration) has approved multiple immunotherapy drugs covering more than 50 types of cancer, which speaks volumes about the importance of immunotherapy in the field of oncology [15–17]. Immunotherapy can be divided into four main categories: immune checkpoint inhibitors, vaccines, percutaneous therapies and non-specific immune boosters [18–21]. While immunotherapy is gaining attention by showing good performance in some populations, there is also a growing awareness of some clinically relevant manifestations. Like surgery, radiation therapy, and chemotherapy regimens, immunotherapy does not benefit all patients, at least in solid tumors where treatment is not as effective as it could be. These factors have been summarized to include, but are not limited to, heterogeneity of antigen expression, poor bioavailability, and an overpowering TIME (tumor immunosuppressive microenvironment), all of which contribute to low response rates. The limited beneficiary population has also become an urgent breakthrough for tumor immunotherapy [22, 23].

**Scheme 1:**
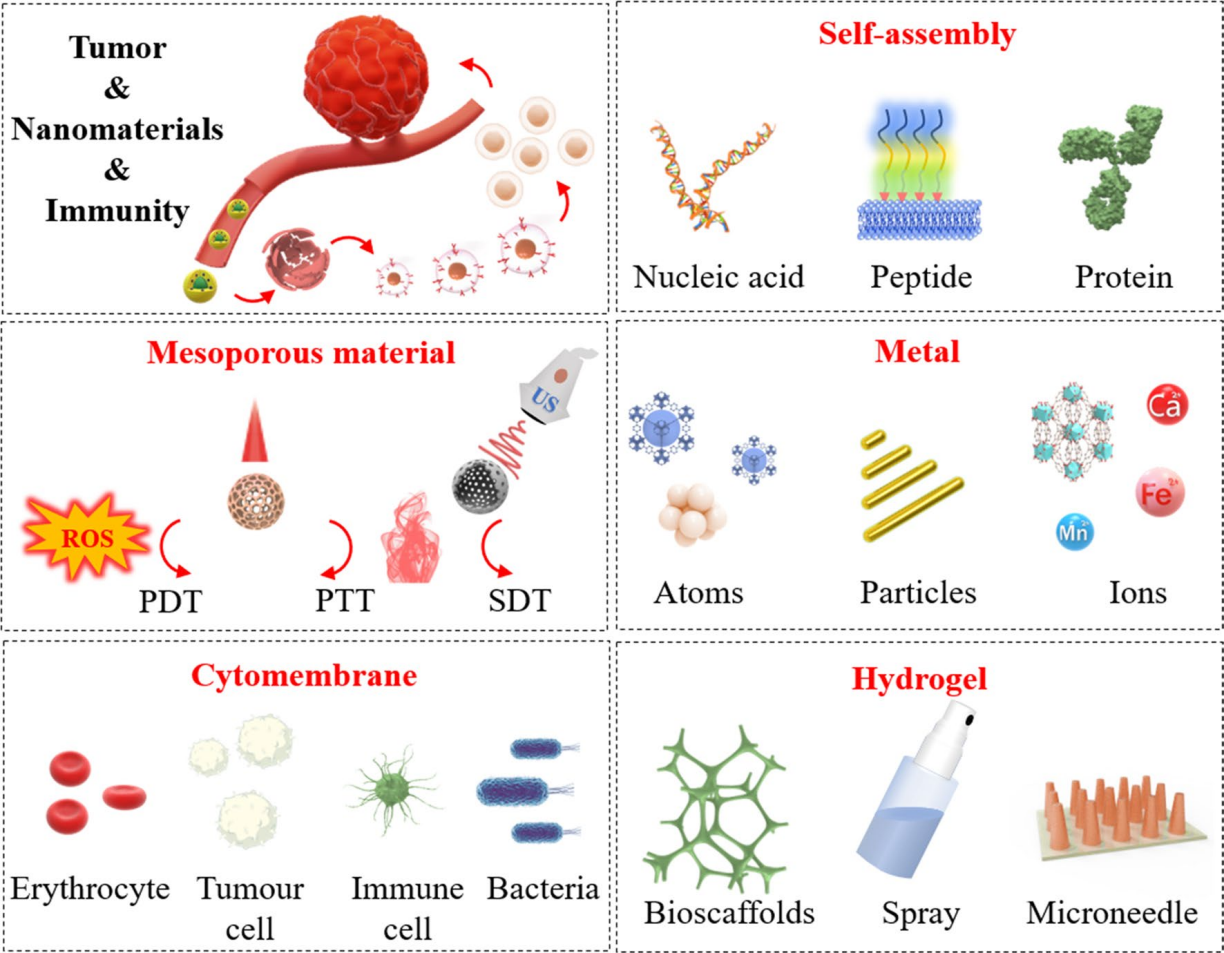
Schematic diagram of cell membrane modified, self-assembled, mesoporous, metallic, and hydrogel nanomaterials in tumor immunotherapy. To enhance anti-tumor efficacy, nanomaterials are introduced as quality companions for immunotherapy

With the continuous optimization of nano synthesis processes, nanobiotechnology application provides viable antitumor immunotherapy solutions [24, 25]. Different synthesis processes and modification schemes determine the diversity of nanomaterials for antitumor immunotherapy. The superiority of this diversity in tumor immunotherapy includes but is not limited to: (i) the response mode and morphological changes of nanomaterials. They can show morphological changes such as dissociation, aggregation or ordered arrangement depending on different environments (pH, temperature, ROS/GSH, etc.) [26, 27]; (ii) Action on different barriers: penetration or adhesion to mucous membranes, penetration of the blood–brain barrier and evasion of the monocyte-macrophage phagocytic system [28, 29]; (iii) Loading of drugs through various forms: self-assembly, electrostatic adsorption, pore loading of mesopores and so on [30–32]; (iv) Targeted therapy: targeting tumor cells, targeting immune cells, targeting subcellular structures such as mitochondria [33, 34]. Here, we review the application of different nanomaterials in tumor immunotherapy, including biological cell membrane modifications, self-assembly, mesoporous, metallic, and hydrogel nanomaterials (Scheme 1). Although these nanomaterials with different compositions have different principles and modes of action, they all ultimately achieve the common goal of tumor immunotherapy.

## Cell membrane modified nanomaterials

The surface properties of nanomaterials play a decisive role in their biological applications. Although EPR (enhanced permeability and retention effect)-mediated passive targeting is widely used in tumor models, the design of any of them requires comparing the affinity of the drug with various proteins in vivo [35–38]. This has led researchers to focus more attention on active targeting. Compared to the traditional surface modification of nanomaterials by physical adsorption and chemical coupling, the introduction of cell membranes ensures the structural stability of nanomaterials in complex environments. Also, it avoids the use of harmful organic solvents. In addition to separating the external environment from the cell, the cell membrane is notably responsible for exchanging and transporting substances and energy. This reciprocal transfer makes it loaded with a large amount of cellular information. Due to their biological origin, modified nanomaterials have good biosafety. And the structure enclosed by the cell membrane provides enough space for drug loading, enabling efficient drug delivery [39, 40]. This section reviews the application of cell membrane-modified nanomaterials of tumor cells, immune cells, erythrocytes, and bacterial origin in tumor immunotherapy.

### Tumor membrane modified nanomaterials

The vigorous development of sequencing technology has led to a deeper understanding of the process of tumor development and the discovery of many immune-related markers of tumors. TAAs (tumor-associated antigens) induce immune cells to recognize and trigger the body's immune response is a common means of tumor immunotherapy [41, 42]. However, the cunning tumor cells also make this scheme ineffective by adjusting the expression of the corresponding proteins in the process. On the one hand, the modification of nanomaterials using tumor cell membranes well ensures the diversity of tumor antigens. On the other hand, the homing ability mediated by surface membrane proteins enhances the targeting of nanomaterials [43, 44]. Moreover, the preparation of the relevant nanomaterials is significantly reduced due to the rapid proliferation of tumor cells and the simple extraction of their cell membranes.

Tumor vaccines are a therapeutic modality that activates tumor recognition by the immune system by providing tumor-associated antigens [45, 46]. However, tumor escape to a single immune target and avoidance of interference from irrelevant antigens in whole cell lysates limit the advancement of tumor vaccines. With this background, Zhang et al. [47] designed the tumor nano-vaccine CpG-CCNP using a protocol of mouse melanoma B16-F10 cell membranes wrapped with the immune adjuvant CpG. Compared to whole cell lysate, CpG-CCNP well inherited TAAs including MART1, TRP2 and gp100 on B16-F10 cell membranes. In vitro experiments, CpG-CCNP was rapidly endocytosed by BMDCs (bone marrow-derived dendritic cells) and activated immune-related cytokines. Among them, the secretion of interleukin-6 (IL-6) and IL-12 were much higher than that of the free CpG group, which also indicates that the encapsulation of the cell membrane facilitated the internalization of the immune adjuvant by antigen-presenting cells compared to free CpG. In vivo experiments, fluorescently labeled CpG-CCNP was administered subcutaneously to mice. The fluorescent signal appeared in the draining lymph nodes and in the adjacent lymph nodes at 1 and 24 h, respectively. These nanoparticles were more frequently taken up by antigen-presenting cells such as dendritic cells and macrophages and were less abundant in the remaining immune cells. CpG-CCNP effectively promoted the maturation of dendritic cells in the draining lymph nodes and stimulated T-cell responses. In the B16-F10 tumor-bearing mouse model, CpG-CCNP provided optimal protection as a tumor vaccine for mice, effectively prolonging median survival, in contrast to CpG-NPs, which had no such effect. To ensure that the use of CpG-CCNP was not compromised by immunosuppression, the team combined CpG-CCNP with anti-CTLA4/anti-PD1. The results showed that the combination strategy inhibited tumor growth and prolonged survival.

The ideal cancer vaccine to achieve efficient anti-tumor effects could have them targeted to immune organs to induce targeted activation in addition to conventional TAAs and immune adjuvants. Among the many immune organs, lymph nodes reside a large number of APCs and T cells, which are the front-runners for antitumor immunotherapy [48, 49]. Sun et al. [50] used B16-F10 tumor cell membranes to wrap aluminum phosphate nanoparticles and CpG to make nanoparticles APMC. Aluminum phosphate is an immune adjuvant with a high safety profile and has been used clinically. In this protocol, binding aluminum phosphate carriers to CpG can trigger cellular immunity. Encapsulation of the B16-F10 cell membrane provides a full range of tumor-associated antigens and ensures the dispersion, mobility, and stability of APMC and, importantly, its lymph node targeting. The results showed that APMC was injected subcutaneously at an appropriate size (60 nm) to facilitate its drainage to lymph nodes. The uptake of TAAs and CpG in APMC by antigen-presenting cells accelerated their maturation. In vivo experiments, APMC stimulated strong cellular immunity, including CD4^+^T cells, CD8^+^T cells, and CTLs, and also promoted the release of immune factors in the spleen and lymph nodes. Ultimately, tumor growth was significantly suppressed, and survival was effectively prolonged after receiving APMC in prophylactic and therapeutic tumor-bearing mouse models.

Although tumor-specific peptides and proteins provide TAAs, the expression profile variability of TAAs limits the application of tumor vaccines in patients with different cancers. Modification of nanomaterials using tumor cell membranes provides a new idea for tumor vaccine development, but how to improve their targeting ability to antigen-presenting cells (APCs) is still a problem to be solved. Zhang et al. [43] used PLGA loaded with the agonist R837, a TLR-7 (anti-toll-like receptor 7) agonist [51], after wrapping the nanomaterials with membranes from B16-OVA cancer cells to obtain NP-R@M. NP-R@M-M was obtained by modifying mannose that can bind specifically to APCs on the surface of NP-R@M. The results showed that NP-R@M-M promoted DC uptake and stimulated DC maturation, which could effectively activate the immune response. In combination with α-PD-1, it showed its powerful therapeutic ability. Similarly, Zhou et al. [52] used cancer cell membranes encapsulated with PLGA nanoparticles R@P-IM loaded with TLR7 agonist R837. Interestingly the cancer cell membranes overexpressed CRT (calreticulin), a signaling protein that induces uptake and activates an immune response in DCs (Dendritic cells) [53, 54], in an in vitro induced manner. The results showed that DCs effectively took up R@P-IM to promote anti-tumor effects. More importantly, the vaccine also activates immune memory to provide long-lasting protection.

### Immune membrane modified nanomaterials

As the cornerstone of the immune system, immune cells permeate tumor immunotherapy in different ways. However, with the evolution of cancer cells, the body's original immune defenses are breached or paralyzed. Boosting or reviving the body's immune system with nanomaterials offers a ray of hope for tumor treatment [19, 55–57]. To ensure that nanomaterials are not recognized by the residual or renegade immune system, it has been proposed that modification of nanomaterials with immune cell membranes can effectively solve this problem.

**Fig. 1:**
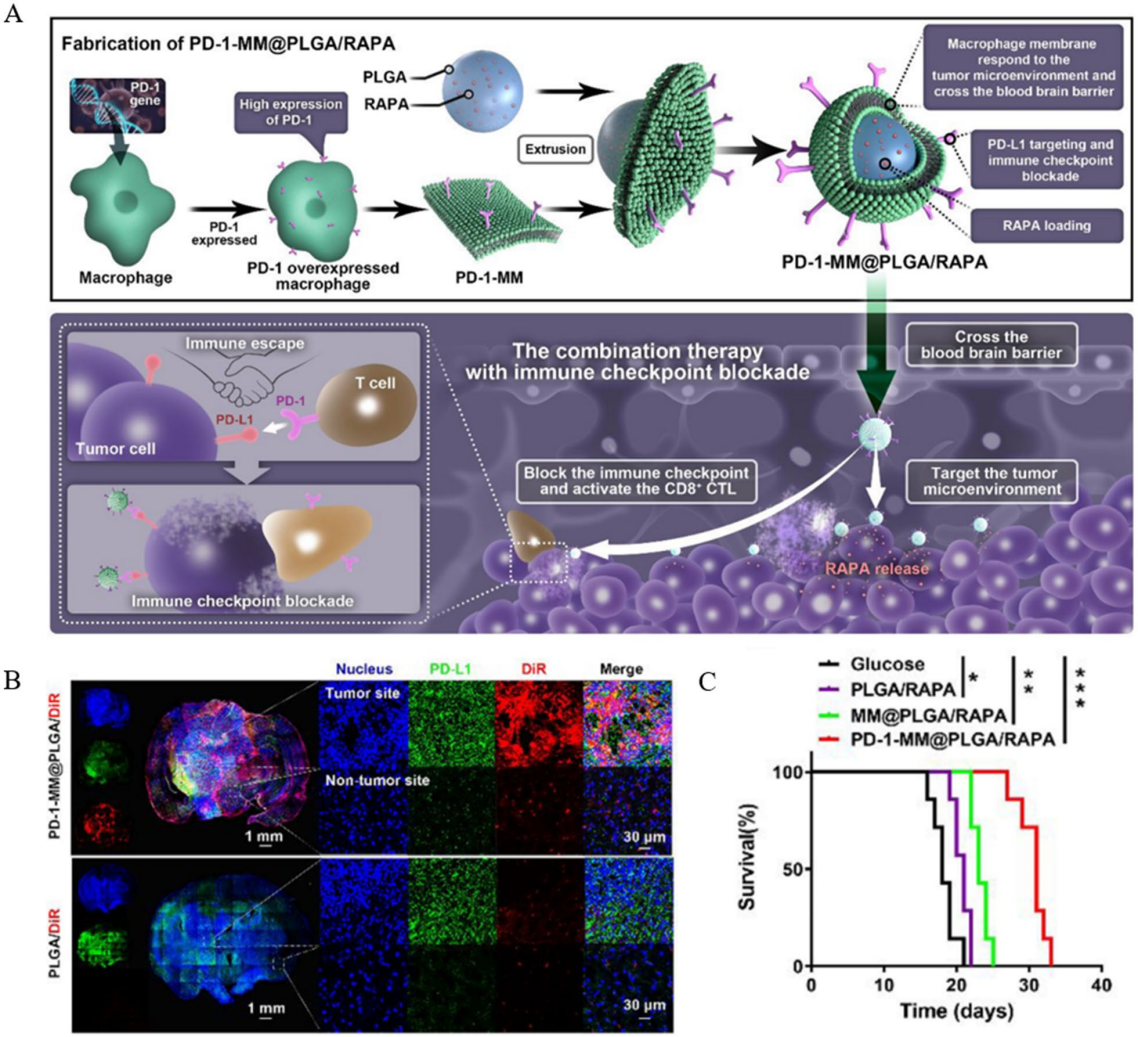
Preparation, mechanism of action and effect of PD-1-MM@PLGA/RAPA. **A** Schematic diagram of the preparation of PD-1-MM@PLGA/RAPA, a macrophage membrane-based nanomaterial, and tumor immunotherapy. **B** Fluorescence images of brain sections 24 h after intravenous injection of PLGA/DiR and PD-1-MM@PLGA/DiR. **C** Kaplan–Meier survival analysis of mice receiving treatments including PD-1-MM@PLGA/RAPA (*p < 0.05, **p < 0.01, ***p < 0.001). Reprinted with permission from Ref. [63]. Copyright© 2022, copyright Yin et al.

GBM (glioblastoma) is a common neurological tumor that is highly malignant and often ends in poor treatment outcomes and prognosis due to the BBB (blood–brain barrier) and TIME [58, 59]. It was found that the binding of PD-1 from TILs (tumor infiltrating T lymphocytes) to tumor cells and TAMs (tumor-associated macrophages) highly expressing PD-L1 inhibited the activation of CTLs, which ultimately led to immune escape [60–62]. Therefore, the investigators used the PD-1/PD-L1 signaling axis as a starting point to improve the immune microenvironment in glioblastoma. Considering the effect of the blood–brain barrier on drug delivery, Wang et al. [63] proposed using macrophage membranes with good trans-blood–brain barrier ability and tumor homing function for nanodrug delivery. PD-1-MM@PLGA/RAPA consists of a PD-1 overexpressing macrophage membrane wrapped with RAPA-loaded PLGA. Using transfection and screening techniques to make macrophage membranes overexpress PD-1, they can compete for PD-L1 binding, allowing T cells to perform their original immune function through the PD-1/PD-L1 axis. The loading of RAPA enhanced the anti-tumor effect by upregulating the ICB response (Fig. 1A). In situ and subcutaneous tumor models of GBM were constructed to examine the mechanism and effect of PD-1-MM@PLGA/RAPA treatment. The results showed that macrophage membrane modification and high PD-1 expression ensured NPs not only reached the brain parenchyma through the BBB effectively but also enriched tumor sites with high PD-L1 expression (Fig. 1B). Markers related to the degree of malignancy were significantly lower in the PD-1-MM@PLGA/RAPA group. Notably, CD8^+^CTLs significantly increased infiltration at tumor sites and enhanced anti-tumor immune response by releasing cytokines such as TNF-α. Ultimately, NPs significantly inhibited the rapid tumor growth and effectively prolonged the survival of tumor-bearing mice (Fig. 1C). This strategy not only solves the problem of glioma, a class of brain tumors that requires delivery across the BBB but also upregulates the ICB response to synergistically enhance the anti-glioma effect, providing a new idea for non-invasive immunotherapy of neurological tumors. DCs occupy a central position in tumor immunity as APCs. Although the tremendous antitumor potential of DCs has been recognized, their action is limited by the immunosuppression of the tumor microenvironment and insufficient infiltration.

On this basis, Cai et al. [64] prepared a delivery platform for iDCs using DCs membranes wrapped with the photothermal agent IR-797 nanoparticles. In vitro experiments, these iDCs, which retained the co-stimulatory signals of MHCI, MHCII, CD80 and CD86, could effectively activate T cells. And the targeting of iDCs was T-cell specific and did not target other immune cells such as macrophages. To confirm that IR-797-mediated PTT can effectively participate in the treatment, NPs were irradiated for 15 min using an 808 nm laser with a controlled temperature of 42–45 °C. The results showed that PTT did not affect the functional regulation of T cells by iDCs. Notably, laser-activated iDCs could inhibit the expression of tumor HSPs (heat shock proteins), and synergistic photothermal treatment effectively induced ICD (immunogenic cell death) based on greater sensitivity to heat stress. In vivo experiments, we artificially constructed tumor models in situ as well as metastasis in both legs of mice. The drug was administered by intratumoral injection and the primary tumor site was irradiated with an 808 nm laser for 30 min at a temperature maintained at 42–45 °C two days later. In the end, satisfactory results were presented: the primary tumor was effectively suppressed, and the growth of distant tumors was controlled, which ultimately significantly improved the survival time. In the analysis of primary tumors in mice, the team found that the number of activated CD8^+^T and CD4^+^T was significantly increased in the iDCs treatment group and promoted the secretion of inflammatory factors including TNF-α, IFN-γ, IL-2 and IL-12p70, which are crucial to the body's anti-tumor immunity.

Although IFN-I-mediated immunotherapy has been a breakthrough therapeutic option, its further development has been limited by poor targeting and toxic side effects [65]. Zhang et al. [66] engineered T cells to express PD-1 at high levels, used their membranes to encapsulate nanoparticles containing the ORY-1001 inhibitor albumin, and finally modified the membrane penetrating peptide on the cell membrane surface to obtain the nanoparticles OPEN. In animal experiments, PD-1-mediated targeting led to nanoparticle aggregation at tumor sites, followed by the intracellular release of lysine-specific histone demethylase 1 (LSD1) inhibitor ORY-1001 by OPEN to upregulate IFN expression. This cascade of reactions allowed the activation and presentation of TAAs by APCs, increased infiltration of total and activated T cells, and ultimately strongly inhibited tumor growth.

Myeloid suppressor cells (MDSCs) impede tumor immunotherapy by suppressing T lymphocyte-mediated anti-tumor immune responses and promoting tumor growth and metastasis [67–69]. Wang et al. [70] prepared pCSs nanoparticles with spongy properties that can adsorb and neutralize responsive cytokines using neutrophil membrane-encapsulated PLGA NPs (poly (lactic acid-hydroxyacetic acid) polymer nanoparticles). Due to the well-inherited properties of neutrophils, pCSs avoided the proliferation of MDSCs and tumor metastasis by adsorbing cytokines [71–73]. In vivo experiments, injection of pCSs into tumor-bearing mice resulted in reduced accumulation of MDSCs in tumors and surrounding lymphoid organs and no compensatory increase in other myeloid subpopulations. The treatment regimen reintroduced the anti-tumor function of T lymphocytes and significantly reduced tumor progression. Moreover, the team combined pCSs with a PD-1 regimen, which not only further inhibited tumor growth but also effectively prolonged the survival of tumor-bearing mice. In summary, immune cells not only play an extremely important role in the fight against tumors but also may become the "traitor" of tumor immunity. Their role in the tumor immune microenvironment can provide new ideas to overcome immunotherapy resistance in clinical settings.

### Erythrocyte membrane modified nanomaterials

The biconcave morphology of erythrocytes is the basis for their excellent deformability and ensures their smooth passage in capillaries. Erythrocytes respond to the body's complex immune system during their 120-day life cycle [74–76]. This is why erythrocyte membrane bionanomaterials are widely used in the biomedical field. On the one hand, normal RBCs (red blood cell) avoid clearance by the immune system due to their unique "cloaking" properties, ensuring the long-lasting retention of their drug load. On the other hand, by taking advantage of the fact that senescent or damaged RBCs are cleared by immune cells, erythrocyte-modified nanomaterials can be targeted to respond to immune cells [77, 78]. Also, the widespread availability of RBCs and the extraction process's simplicity make them promising for drug delivery systems at large.

Tumor immunotherapy is ineffective in most solid tumors, and the lack of immunogenicity is an essential factor that cannot be ignored. These tumors, defined as "cold tumors" due to immune deficiency or absence, result from multiple factors, including irregular distribution and malformation of tumor vasculature [79–81]. This phenomenon also leads to hypoxia in the tumor microenvironment exacerbating the tumor immunosuppressive microenvironment. NO (nitric oxide), which regulates the normalization of tumor vasculature, can reverse immunosuppression by improving tumor hypoxia [82–84]. However, the available NO molecules and the donor suffer from poor stability. Therefore, Tian et al. [85] used the NO macromolecule donor SNO, the NIRII photothermal agent IR1061, and the IDO-1 (indoleamine 2,3-dioxygenase 1) inhibitor 1-MT (1-methyltryptophan) to self-assemble into nanospheres and then wrapped them with erythrocyte membranes to finally obtain RBCm/PAAV-SNO/1-MT + IR1061. The erythrocyte membrane provides a safe passage for the entire nanomaterial to the tumor site, ensuring long-lasting retention of the nanomaterial in the body to enhance needle accumulation at the tumor site. When the NPs reach the tumor site, IR1061 raises the tumor temperature by photothermal conversion under the action of NIR-II. The elevated temperature, on the one hand, triggers S-NO bond breaking to induce the precise release of NO and 1-MT, and on the other hand, recruits effector T lymphocytes to accumulate in the tumor tissue to mobilize anti-tumor immunity by inducing ICD. The NPs were injected intravenously into 4T1 tumor-bearing mice on days 1, 4, and 7, respectively, and laser-based irradiation was used to verify their effectiveness 24 h after administration. The results showed that combining RBCm/PAAV-SNO/1-MT + IR1061 with laser irradiation resulted in a 66.7% survival rate and a significant reduction in the mean tumor size on day 21, and the same good results were still observed on day 48. It was also found that the tumor vascular distribution was more orderly after RBCm/PAAV-SNO/1-MT + IR1061 combination laser irradiation due to the effect of NO, suggesting normalization of tumor vasculature. Importantly, this strategy induced infiltration of CD8^+^CTL at the tumor site, and INF-γ and TNF-α were also significantly enhanced. The regimen of RBCm/PAAV-SNO/1-MT + IR1061 combined with laser irradiation achieved tumor immunotherapy from multiple aspects and broke the bottleneck of low tumor immunogenicity.

**Fig. 2:**
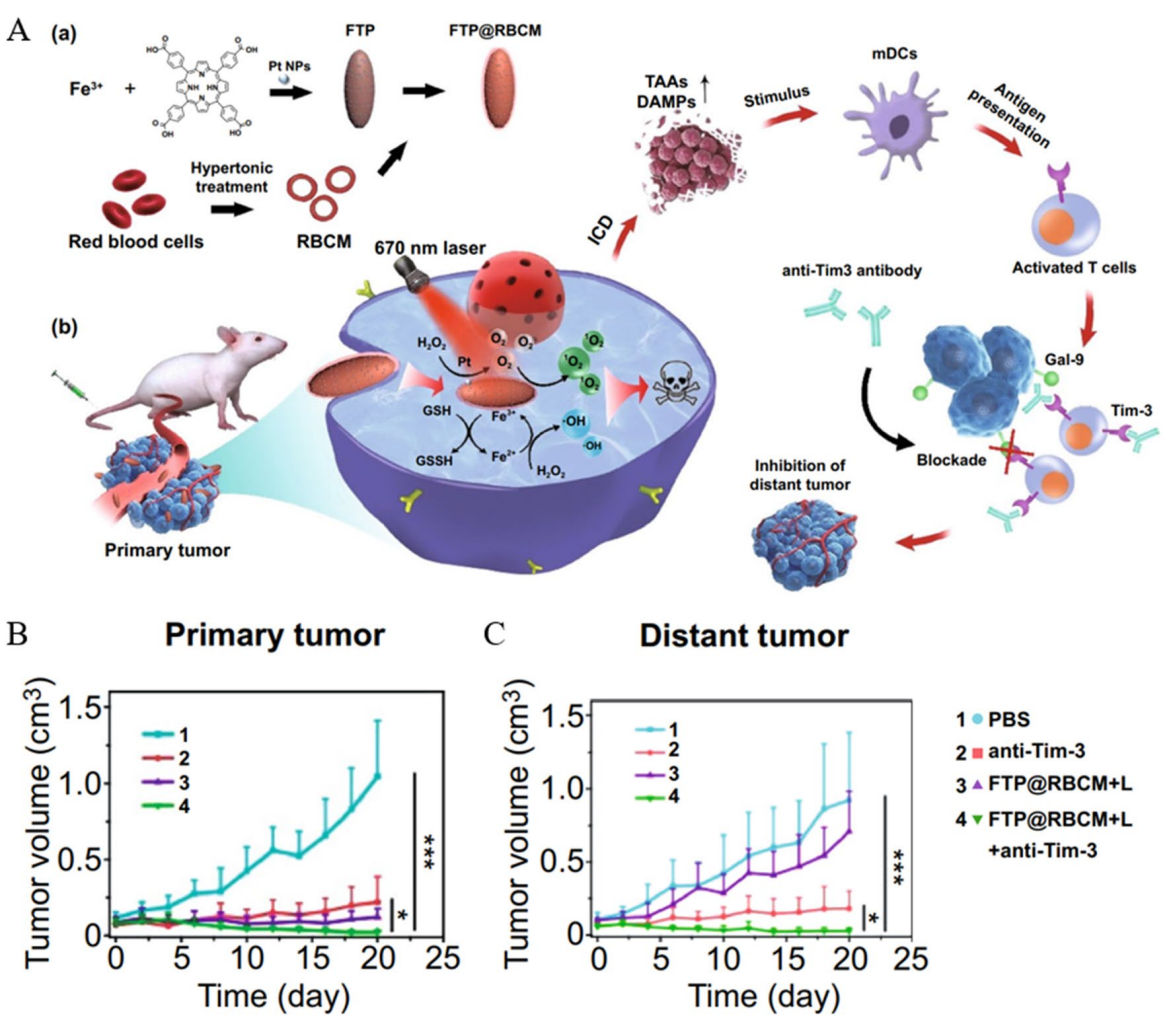
FTP@RBCM-ICG antitumor efficacy. **A** Schematic diagram of FTP@ RBCM-ICG preparation and mechanism of action. **B**, **C** Primary and distant tumor volumes of tumor-bearing mice treated with PBS, anti-Tim-3, FTP@RBCM-ICG, and FTP@RBCM-ICG + anti-Tim-3 (*p < 0.05, ***p < 0.001). Reprinted with permission from Ref. [89]. Copyright© 2022, copyright Li et al.

Exogenous delivery or endogenous induction of ROS is considered an effective option to eradicate cancer cells. However, tumor hypoxia and difficulty in Fenton reaction induction limit its application. The practical component for inducing ROS production in the Fenton reaction is Fe^2+^ rather than Fe^3+^, and if recycling of Fe^2+^ is achieved, it is crucial for efficient induction of ROS production [86–88]. Yao et al. [89] proposed to use a core of iron porphyrin-based MOF, doped with Pt nanoparticles and then modified with erythrocyte membranes in the outermost layer to obtain FTP@RBCM as an enforcer of the radical storm. The protocol also combines the novel immune checkpoint blocking scheme Tim-3, a molecule expressed in various immune cells to regulate the immune response. In vitro assay of catalytic activity, the reduction of Fe^3+^ to Fe^2+^ by GSH in MOF resulted in a significant decrease in GSH content. Due to the presence of Pt, H_2_O_2_ in cells can be effectively catalyzed to O_2_, solving the problem of cellular hypoxia (Fig. 2A). The ROS detection probe showed a solid green fluorescent signal in cells treated with FTP@RBCM and laser irradiation under hypoxic conditions, illustrating the effectiveness of FTP@RBCM as a ROS storm enforcer. Before in vivo experiments, hemolysis assays showed less than 3% hemolysis in FTP@RBCM at TCPP concentrations up to 80 ug mL^−1^, ensuring good biocompatibility. ICG (indocyanine green) labeling of NPs (FTP@ RBCM-ICG) was used to probe the biodistribution. The fluorescence signal at the tumor site peaked at 5 h after intravenous injection. It was higher than FTP-ICG, interpreted as RBCMs enhancing aggregation at the tumor site by prolonging in vivo residence. More signal accumulation at the tumor site was observed on fluorescence imaging of isolated organs and tumors. The tumor volume of mice was significantly reduced after NPs combined with laser irradiation treatment, which was attributed to the FTP@RBCM-induced free radical storm with multiple catalytic activities in TME. Moreover, tumor tissues showed high expression of CRT and a significant increase in DCs maturation rate in inguinal draining lymph nodes of mice. To evaluate the efficacy of the combination with anti-Tim-3 antibody, a bilateral Hepa1-6 tumor model including primary and distal tumors was constructed. The results showed that although FTP@RBCM combined with laser irradiation had an excellent inhibitory effect on primary tumors, it was ineffective for distant tumors. In contrast, FTP@RBCM combined with laser irradiation + anti-Tim-3 showed good inhibition of both primary and distant tumors (Fig. 2B, C), with significant increases in CD8^+^T in primary and distant tumors and spleen and a 100% survival rate in mice after 55 days of treatment. This implies that a systemic immune response was activated under a synergistic strategy, and long-term immune memory was developed.

In addition to their standard transport functions, erythrocytes also function as immune "scouts." They drive oxidative stress against foreign substances present them to APCs [90]. Considering erythrocytes' immune function, using them as carriers for antigen delivery effectively induces immune responses and reduces toxic organismal effects. However, since the close contact of the endothelium in the pulmonary capillaries can squeeze the erythrocytes and lead to the shedding of surface drug carriers, the design of a lung-escaping erythrocyte carrier antigen delivery system has a vast prospect. Mitragotri et al. [91] used ovalbumin-coated polystyrene carboxylates to form nanoparticles and loaded them onto the erythrocyte surface at a ratio of 300:1 to obtain EDIT. DIT ensured both spleen-directed delivery of immune material and resistance to lung clearance. In in vivo experiments, EDIT efficiently activated T cells and delivered a satisfactory result for immunotherapy with all remaining tumor-free. Le et al. (35430766) used EV (extracellular vesicles) of RBCs as a vehicle to target tumor cells with RIG-I agonists that can lead to type I interferon release and immune activation. The results showed that RBCEVs effectively inhibited tumor growth and metastasis by increasing immune cell infiltration and CD8^+^T cell immune response in situ and metastatic tumors.

### Bacterial membrane modified nanomaterials

Attenuated bacterial strains are an effective tool widely used in oncology treatment. For example, BCG for bladder cancer has a history of almost a century [92, 93]. Despite the clinical importance of bacterial-derived therapeutic regimens, the exact mechanisms involved remain unclear. However, there is consensus that they can achieve tumor treatment by modulating immune cells such as CD4^+^T, CD8^+^T cells, Treg (regulatory T cells) and TAMs.

**Fig. 3:**
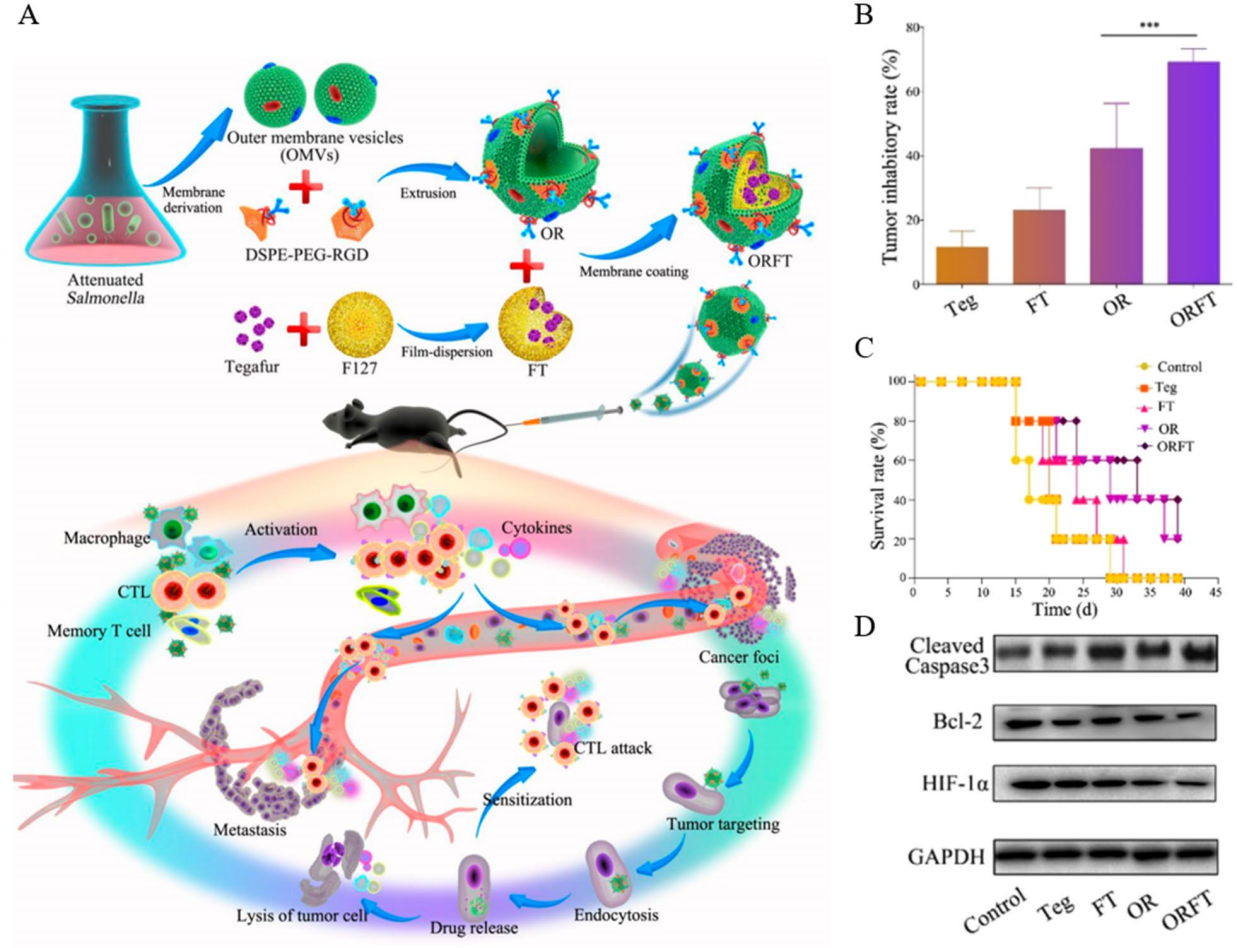
Anti-tumor efficacy of ORFT. **A** Schematic representation of the preparation and mechanism of action of ORFT derived from OMVs. **B** Tumor inhibitory rate (%) after treatments with ORFT et al. (mean ± SD, n = 5) **C** Survival rate of B16-F10 tumor-bearing mice after treatment with ORFT and other regimens. **D** Western blot analysis of cleaved caspase-3, Bcl-2 and HIF-lα protein expression in tumor tissues after receiving the corresponding treatments (***p < 0.001). Reprinted with permission from Ref. [99]. Copyright© 2020, copyright Chen et al.

Salmonella, a strain that can replicate in living and necrotic tissues, has been used to target deep hypoxic tissues in tumors [94–96]. However, its low tumor suppression rate and dose dependence limit its clinical application. It was found that Gram-negative membrane-released bacterial OMVs (outer membrane vesicles) are superior immunotherapeutic agents because they have most of the immunogenic membrane-associated and surface-associated components of their parental bacteria, with smaller size (20–250 nm) and safer properties. Similarly, OMVs have good tumor targeting ability and are often ideal carriers for oncology therapeutics [97, 98]. Ping et al. [99] wrapped Tegafur, a prodrug of 5-FU, with F127 and subsequently wrapped it with OMVs functionalized with PEG (polyethylene glycol) and tumor-targeting peptide RGD to obtain ORFT. Tegafur, a prodrug of fluorouracil (5-FU), was used as a drug candidate to enhance the immunostimulatory capacity of OMVs. In addition to killing tumors in the form of apoptosis, 5-FU also enhances the sensitivity of cancer cells to CTL. More importantly, 5-FU can eradicate bone marrow-derived suppressor cells with immunosuppressive function. However, the immunomodulation involved in 5-FU takes effect after immune activation, so the choice of 5-FU prodrug is more relevant (Fig. 3A). In vivo experiments, tumor growth was significantly inhibited up to 70% after 21 days by fractionated intravenous injection of ORFT into tumor-bearing mice (Fig. 3B), and the survival rate of the mice was improved (Fig. 3C). Pleasingly, the structure and function of major organs were unchanged in H&E staining and blood biochemical assays, indicating the superior biocompatibility of ORFT. In vivo experiments selected B16-F10, which has a high degree of malignancy as well as metastatic ability, to construct a tumor-bearing mouse model. Compared with the control group, lung metastasis was significantly inhibited in mice after treatment with ORFT. And the expression of apoptosis-related molecules cleaved caspase-3 was significantly highest in the tumors, while Bcl-2 and HIF-lα expression were significantly lowest (Fig. 3D). The excellent performance of ORFT in tumor immunotherapy and anti-metastasis gives OMV a remarkably high recognition as a drug delivery vehicle and promotes the application of bacterial membranes in anti-tumor immunity.

Fellow stars of tumor immunotherapy, the immune checkpoints PD-1 and PD-L1 have achieved encouraging efficacy in some tumors. However, due to the low responsiveness of this strategy and its toxic side effects, researchers are continuously developing and modifying them [100, 101]. Guo et al. [102] constructed NPs with PD-1 self-blockade using LyP1 tumor-targeting peptide-modified bacterial outer membrane vesicles (LOMV) wrapped with PD-1 plasmids. As models, in vivo experiments were performed using 4T1 breast cancer, B16-F10 melanoma, and CT26 colorectal carcinoma hooded mice. The results showed that good tumor suppression was achieved by intravenous injection of NPs in both the hormonal mouse models with high immunogenicity and low response rate. In this process, there is not only the reactivation of CTL after PD-1/PD-L1 pathway blockade but also the induction of CTL differentiation into central memory T cells by OMVs to generate long-term immunity.

Moreover, OMVs enhance the infiltration of CTL and NK cells in tumor tissues, achieving a multifaceted anti-tumor effect [103]. Wang et al. [104] proposed the use of OMVs to wrap β-CD and ADA-modified GNPs to obtain M-CD-GNPS and M-ADA-GNPs, respectively, which undergo degradation of OMVs under phagocytosis of immune cells and subsequently drive the self-assembly of GNPs under β-CD-ADA host–guest interactions. In vivo experiments, laser irradiation was administered for 5 min at 1 h and 4 h after tail vein injection of NPs, in concert with aPD-L1 treatment. The results showed that NPs + laser + α-PD-L1 achieved the best tumor suppression effect and effectively prolonged the survival of mice compared with the rest of the experimental groups. Similarly, Gan et al. [105] constructed CuS-OMV using E. coli-derived OMV wrapped with CuS with high photothermal conversion efficiency. In vivo experiments revealed that CuS-OMVs had good tumor targeting properties, which laid the foundation for subsequent PTT-targeted therapy. CuS acts as an excellent photothermal conversion agent to inhibit tumor cells at high temperatures under NIR-II irradiation. CuS-OMVs can not only promote DC maturation and CD8^+^T cell activation by inducing ICD in tumor cells but also induce M1 polarization and thus reverse the TIME.

Nie et al. [106, 107] has designed a "plug-and-play" universal tumor vaccine platform using genetic engineering techniques to fuse tumor antigens with the ClyA protein on the surface of OMVs. This vaccine vector relies on a small size to enable lymph node drainage. Importantly, this strategy allows for "customized" tumor immunotherapy due to the wide range of tumor antigen sources. In contrast to the OMV delivery platform, the team also proposed using bacterial endosomes that activate TLR1/2/6 as a delivery platform for tumor-specific antigens [108]. Unlike conventional immune adjuvants, this protocol avoids undesirable side effects like factor storms. Moreover, it showed good tumor suppression in several mouse tumor models and prevented recurrence by inducing memory T cells, thus promising clinical application.

## Self-assembled nanomaterials

Self-assembly is the spontaneous formation of ordered structures by basic structural units. Their interactions are based on non-covalent bonds rather than the simple superposition of substances [32, 109]. The use of such structural changes facilitates their bioregulation in vivo. For example, small molecule structures quickly pass through barriers such as tumors to reach deep tissues but suffer from the problem of being easily removed without long-lasting retention. Conversely, nanomaterials with larger volumes are less likely to be cleared by the body but are less permeable to tissues. The researchers delivered drugs in the form of small molecules that self-assemble into nanoparticles after penetrating tissues to achieve therapeutic goals with an "easy-in, hard-out" strategy. There are many other options to achieve therapeutic goals by self-assembling drugs with conformational changes [110–113]. This section will review the application of nucleic acid, peptide and protein-based self-assembly strategies in tumor immunotherapy.

### Nucleic acid-based self-assembled nanomaterials

The biological macromolecule nucleic acid is obtained by polymerizing nucleotides consisting of three molecular linkages: nitrogenous bases, ribose or deoxyribose, and phosphate. Nucleic acids are not only essential components of cells but also contribute to biological processes such as growth, evolution, inheritance and mutation of organisms. Researchers have realized the importance of these endogenous substances in tumorigenesis, metastasis, and drug resistance and have proposed nucleic acid-based therapeutic strategies. Among them, nucleic acid-based self-assembled nanomaterials have gained significant attention because of their excellent biocompatibility, precise recognition, and easy synthesis [114–117]. Materials biologists have made them widely used for targeted delivery, cell imaging and other fields through different assembly forms.

Recent studies have found that radiotherapy, which has an absolute place in tumor treatment, triggers ICD while killing the DNA of tumor cells, thus inducing cell necrosis and apoptosis [118–120]. However, this source of ICD is insufficient to induce a potent immune response. Based on these limitations, Tan et al. [121] designed ligand-driven self-assembly of metal ions and DNA into MXFs nanomaterials. This versatile synthesis method ensures the size and crystal structure of nanomaterials in the range of tens to hundreds of nanometers. In vitro assays, Hf-CpG MXF significantly enhanced radiotherapy-induced DNA damage and cell differentiation of mature DCs, while significantly upregulating the expression of the critical ICD molecule calreticulin. In CT26 tumor-bearing mice, radiotherapy combined with Hf-CpG MXF significantly inhibited tumor growth and prolonged the survival time of mice. In contrast to local radiotherapy alone, this regimen effectively suppresses metastatic tumors. This was attributed to radiotherapy and combined Hf-CpG MXF stimulating DC cell maturation and induced infiltration of CD8^+^T and CD4^+^T cells in the distal tumor tissue. Encouragingly, the regimen showed tumor vaccine efficacy by inducing long-term immune memory effects.

The antigen-specific presentation of APCs to T cells is critical for activating CD8^+^T cells for tumor immunotherapy [122, 123]. Considering that the assembly of biomolecules on living cells may affect the function of receptor proteins, Liu et al. [124] implemented natural APCs on LC-aAPCs in a bottom-up self-assembly pathway that mimics the distribution of T cells activation ligands to promote T-cell activation. Lymphocytes still had good homing ability after receiving this lipid-DNA-mediated self-assembly. LC-aAPCs not only efficiently expanded T cells in vitro experiments but also exerted excellent efficacy in vivo experiments. In vivo experiments, LC-aAPCs were injected into mice and found to accumulate mainly in peripheral lymphoid organs and activate specific T cells. A validation study using tumor-bearing mice expressing OVA antigen in melanoma as the models. LC-aAPCs, in combination with α-PD1, achieved potent tumor suppression by increasing CD8^+^T cells and significantly prolonged the survival time of tumor-bearing mice.

The classical antitumor drug DOX (doxorubicin) was found to regulate tumor immunity by inducing ICD and inducing tumor cell death in the form of apoptosis and ferroptosis [125–127]. Unfortunately, this process was also accompanied by the upregulation of immunosuppressive molecules such as PD-L1, which counteracted the effect of ICD. Yang et al. [128] used π–π stacking of PEG-hyd-DOX to self-assemble with siRNA (small interfering RNA) of PD-L1 to form PEG@D:siRNA. After PEG@D:siRNA entered the cells, DOX was activated by the action of lysosomes to induce ICD effectively. This process was not accompanied by an increase in PD-L1 expression, which was attributed to the effective regulation of the siRNA pair of PD-L1. The proposed scheme allows the researchers to expand their ideas to other immune targets. Moreover, siRNA delivery cannot be limited to a single target while ensuring effective loading. Besides, the stability of the nucleic acid delivery system determines the efficiency and even the success or failure of the treatment. Caruso et al. [129] loaded Y-CpG formed by assembling Y-type DNA single strands onto CaCO_3_ particles and cross-linked them using DNA double-stranded linkers. The dissolved CaCO_3_ is then used to obtain DNA nanocapsules with CpG motifs and uniform size. Unlike ssDNA (single-stranded DNA) and Y-CpG modules, the DNA capsules maintain good stability in the extracellular environment and provide a structural basis for subsequent immunotherapy.

### Peptide-based self-assembled nanomaterials

Between 1920, when the world's first peptide insulin was discovered, and 2021, more than 100 peptides have been approved for clinical use. This substance formed by condensing amino acids can perform critical biological functions in the body with its independent structure or through the arrangement of relationships to form proteins. Since peptides are involved in numerous processes such as growth, development and metabolism in the human body, this has led to the development of nanomaterials based on peptides for a wide range of biomedical applications [130–132]. The molecular weight of peptides is about 180–5000 D, and they can be divided in terms of size into small peptides and oligopeptides with molecular weights of 180–1000 D and large peptides with molecular weights of 1000–5000 D [133, 134]. In terms of function, the properties of amino acids, the sequence of amino acids and the shape of peptides determine their biological roles. As a rule, peptides are derived from cellular, natural plant or chemically synthesized pathways. Due to their high biosafety and modifiability, combining them in different forms into nanomedicines or carriers is the mainstream development direction [135–137]. Undoubtedly, self-assembly is the most striking among them, and their delicate design allows them to form into spheres, rods or nets, etc. spontaneously.

ICB breaks the immune defense barrier of tumor cells and has good efficacy in malignant tumors, including breast cancer and non-small cell lung cancer. Among them, therapeutic regimens targeting the PD-1/PD-L1 signaling axis are also being explored [138, 139]. Still, the conventionally used PD-L1 antibodies are difficult to achieve efficient tumor penetration due to their large size, thus becoming a vital issue for ICB to break through in solid tumor therapy. PD-L1 peptide with a smaller size is a strong candidate for ICB therapy due to its excellent tumor penetration ability [140, 141]. Wang et al. [142] developed a click reaction-assisted peptide immune checkpoint blockade strategy. The DBCO-modified targeting peptide (TP) utilizes good tumor permeability and targeting to bind well to PD-L1, which is highly expressed in tumor cell membranes. Subsequently, an azide group-modified assembly peptide (AP) was introduced. The AP peptide was self-aggregated to form a fibrous structure under the click reaction mediated by the azide group and DBCO. In vitro experiments, the nanofibrous structures formed by the self-assembly of Cy-labeled TP-AP still had a fluorescent signal at 12 h compared to Cy-labeled TP, effectively prolonging the occupancy time to PD-L1. The same phenomenon was also shown in the in vivo experiments, where Cy-labeled TP-AP had a stronger and longer-lasting fluorescence signal at the tumor site, which had a crucial impact on its anti-tumor effect. After four doses administered once every 2 days, the CRICB regimen effectively increased CD8^+^T-cell infiltration in tumor tissue compared to the PD-L1 antibody and TP groups, resulting in a 1.5 and 2.1-fold increase in TGI (tumor growth inhibition) values and a significant increase in survival time. Notably, CRICB had no significant toxic side effects, unlike the PD-L1 antibody group, where mouse death was observed, and massive hemorrhage was observed in kidney and lung tissue sections.

**Fig. 4:**
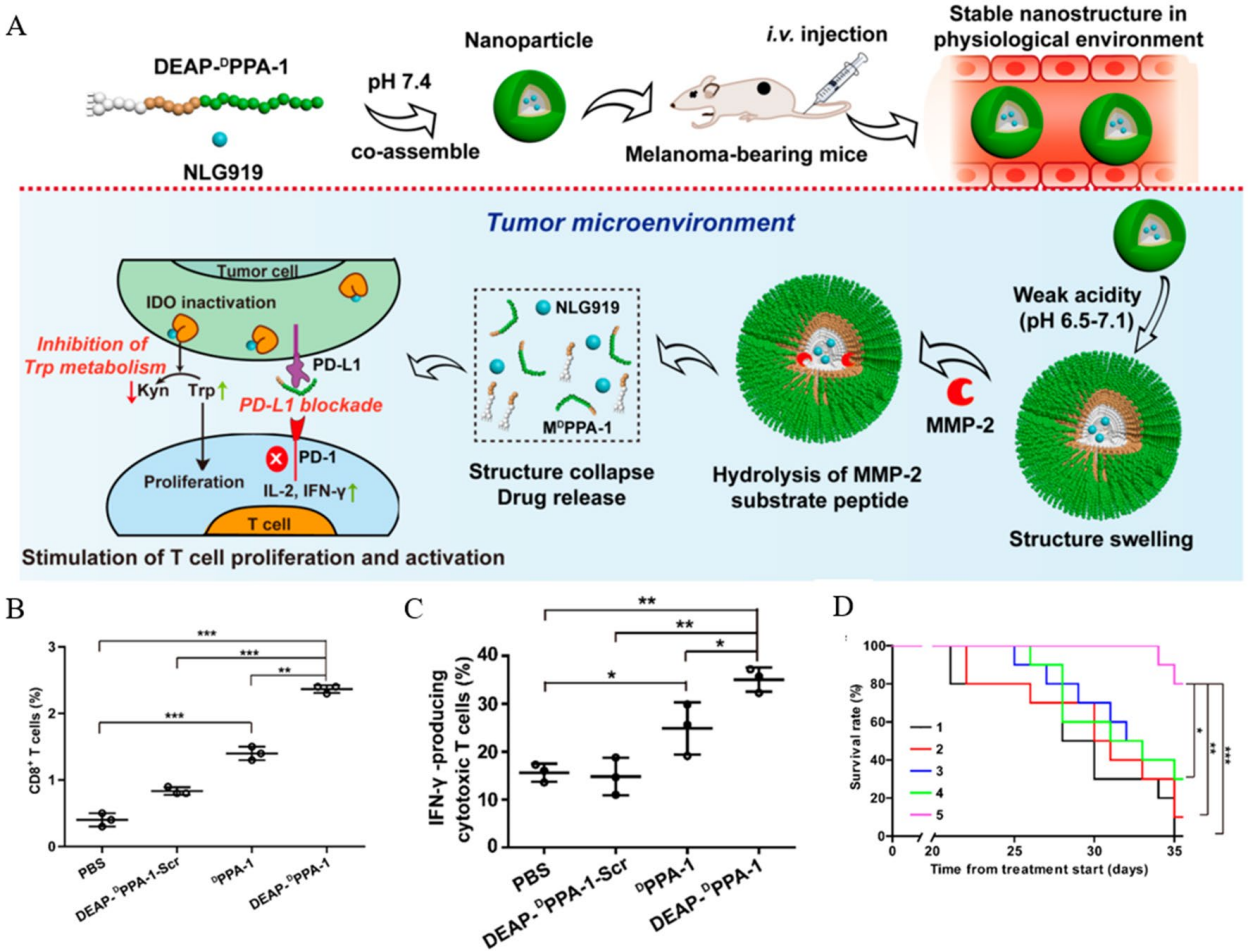
Anti-tumor efficacy of NLG919@DEAP-^D^PPA-1. **A** Schematic representation of the self-assembly process of NLG919@DEAP-^D^PPA-1 and its mechanism of action. **B**, **C** Percentage of CD8^+^T and IFN-γ-producing cytotoxic T cells in tumors after different treatment regimens. **D** Survival rate of B16-F10 tumor-bearing mice after different treatment regimens. (Treatment groups: 1: DEAP-^D^PPA -1-Scr; 2: NLG919; 3: NLG919@DEAP-^D^PPA-1-Scr; 4: DEAP-^D^PPA-1; 5: NLG919@DEAP-^D^PPA-1, *p < 0.05, **p < 0.01, ***p < 0.001). Reprinted with permission from Ref. [143]. Copyright© 2018, copyright Cheng et al.

In conclusion, the peptide self-assembly-mediated therapeutic strategy showed a good immune checkpoint blockade effect, thus expanding its biomedical application. Similar to the immune-related molecule PD-L1 overexpressed in solid tumors is the IDO enzyme, which degrades l-tryptophan to l-kynurenine. The high expression of IDO enzymes inhibits T cell proliferation and activity. Nie et al. [143] used DEAP molecules and MMP-2 peptide substrates to modify PD-L1 antagonistic ^D^PPA-1 peptide, and the resulting DEAP-^D^PPA-1 assembled with IDO inhibitor NLG919 to form NLG919@DEAP-^D^PPA-1. The weak acidic state of TME leads to a decrease in the hydrophobicity of DEAP, allowing the loosening of NPs. At the same time, the final disintegration of NPs is attributed to the hydrolysis of their substrate peptides by MMP-2 (Fig. 4A). In vivo experiments, NLG919@DEAP-^D^PPA-1 inhibited tumor growth in B16-F10 tumor-bearing mice by inducing T cell proliferation and activation (Fig. 4B, C) and significantly prolonged mouse survival (Fig. 4D).

Affected by many factors, such as environmental pollution and smoking, lung cancer has a very high incidence rate worldwide. Despite various treatment options, the 5-year survival rate of advanced lung cancer has not improved significantly, which poses a significant challenge to public health expenditure. It has been found that this refractoriness is closely related to the TIME in lung cancer [144–147]. In this microenvironment, TAM influences tumor immunotherapy in a substantial proportion. Under normal conditions, macrophages process and present foreign material, including tumors, to T cells by engulfing them. However, as tumors evolve, their surface avoids phagocytosis by expressing CD47 "Don't eat me" signals in combination with SIRPα (signal regulatory protein-α) on macrophages [148–151]. Jin et al. [152] designed and constructed a peptide-based MMP-2 (matrix metalloproteinase-2)-responsive CD47-blocking strategy, TMCB, for tumor immunotherapy. This LMY1 peptide consists of a hydrophilic module SSGG, a tailoring motif PLGVRG containing an MMP-2 responsive sequence, a self-assembly module KLVFFC, and a CD47 targeting module. In the presence of the targeting peptide, LMY1 recognizes CD47, which is highly expressed in tumor cells and cleaved by MMP-2, which is highly expressed in TME. The remaining peptide fragments self-assembled on the cell membrane to form stable nanofibers that effectively inhibited the "Don’t eat me" signal of CD47. In vitro assays, the formation of distinct nanofibrils of LMY1 due to the action of MMP-2 was observed by TME. In vivo experiments showed that after the fluorescently labeled LMY1 entered the tumor-bearing mice through the tail vein, a significant fluorescence signal was detected at 0.5 h and peaked at the 8th hour, which lasted until 120 h. This indicates that the nanofibrous structure formed on the cell membrane has an excellent residual ability to mask CD47 molecules for a long time. Based on this, α-PDL-1 was combined to assess its anti-tumor ability in LLC-bearing mice. The results showed that the LMY1 + α-PDL-1 group had the most significant anti-tumor effect compared to other experimental groups, and there were no abnormalities in blood biochemical parameters. This suggests that the LMY1 strategy has high biosafety while achieving a funny antitumor effect. However, it should be noted that the in vivo study of this regimen did not validate indicators of relevant immune cells, including T cells.

### Protein-based self-assembled nanomaterials

Proteins are essential components that makeup cells and tissues in living organisms. This organic macromolecule is not only involved in critical life activities but is also the material basis of tumor cells. Therefore, researchers have focused on relevant proteins involved in tumor growth and regulation and developed a series of protein-based therapeutic solutions. The proteins can spontaneously form morphologies, including loops and cages, and form structures such as fibers and microtubules through the assembly [153–156]. These explorations and discoveries have laid a good foundation for developing protein-based self-assembled nanomaterials and facilitated their application in biomedicine, especially in tumor immunotherapy.

Restoration of anti-tumor immune response can achieve long-term immune surveillance compared to direct killing of tumor cells. However, the TIME, dominated by TAMs, constrain immunotherapy in solid tumors. Therefore, the dismantling of TIME can be achieved by depletion or "re-education" of TAMs [157–159]. Zhou et al. [160] constructed a self-assembled bionanocyte (nano-RBC) system V(Hb) conjugated with Hb-PCL (hemoglobin-poly(ε-caprolactone)) and eventually obtained V(Hb)@DOX after loading DOX. TEM shows that the average V(Hb) size is about 120 nm and has good stability in a neutral environment, in contrast to rupture disintegration in an acidic environment. Hb of V(Hb)@DOX targets M2-type TAMs via CD163 surface receptors after binding to Hp (haptoglobin) in the body plasma. In addition, O_2_ released from Hb ameliorated tumor tissue hypoxia and inhibited the recruitment of M2 types promoting tumor immune response. In vivo experiments, V(Hb)@DOX reversed TIME by the above mechanism. It downregulated immune-negative molecules, including PD-L1, IL-10, and TGF-β, while increasing the immunostimulatory factor IFN-γ and enhancing the CTL response. Ultimately the V(Hb)@DOX strategy effectively inhibited tumor growth in hormonal mice and established a robust immune memory response.

Also, based on the self-assembly of Hb, Sun et al. [161] proposed to combine two protein monomers, Hb and Gox, into multimeric protein superstructures, Hb@GOx NPs, using a self-assembly strategy. On this basis, RBC@Hb@GOx NPs with good biocompatibility were prepared by modifying them using red blood cell membranes with the ability to span BBB. These NPs with a final size of about 50 nm achieved tumor cell killing in vitro by generating a large amount of ROS and effectively promoted the cell membrane translocation of CRT and the release of HMGB1 (high mobility group protein 1). However, in vivo experiments are crucial to examine the antitumor efficacy of RBC@Hb@GOx due to the specific nature of the BBB present in GBM. ICG-labeled RBC@Hb@GOx NPs were injected intravenously into in situ U87MG glioma-bearing mice. The results showed that 12 h after injection, the NPs effectively crossed the BBB to display fluorescent solid signals at the tumor site. This phenomenon persisted until 72 h after injection, demonstrating the long-lasting accumulation of RBC@Hb@GOx NPs at the tumor site. The final RBC@Hb@GOx strategy effectively inhibited the growth of GBM and prolonged the survival time of tumor-bearing mice.

**Fig. 5:**
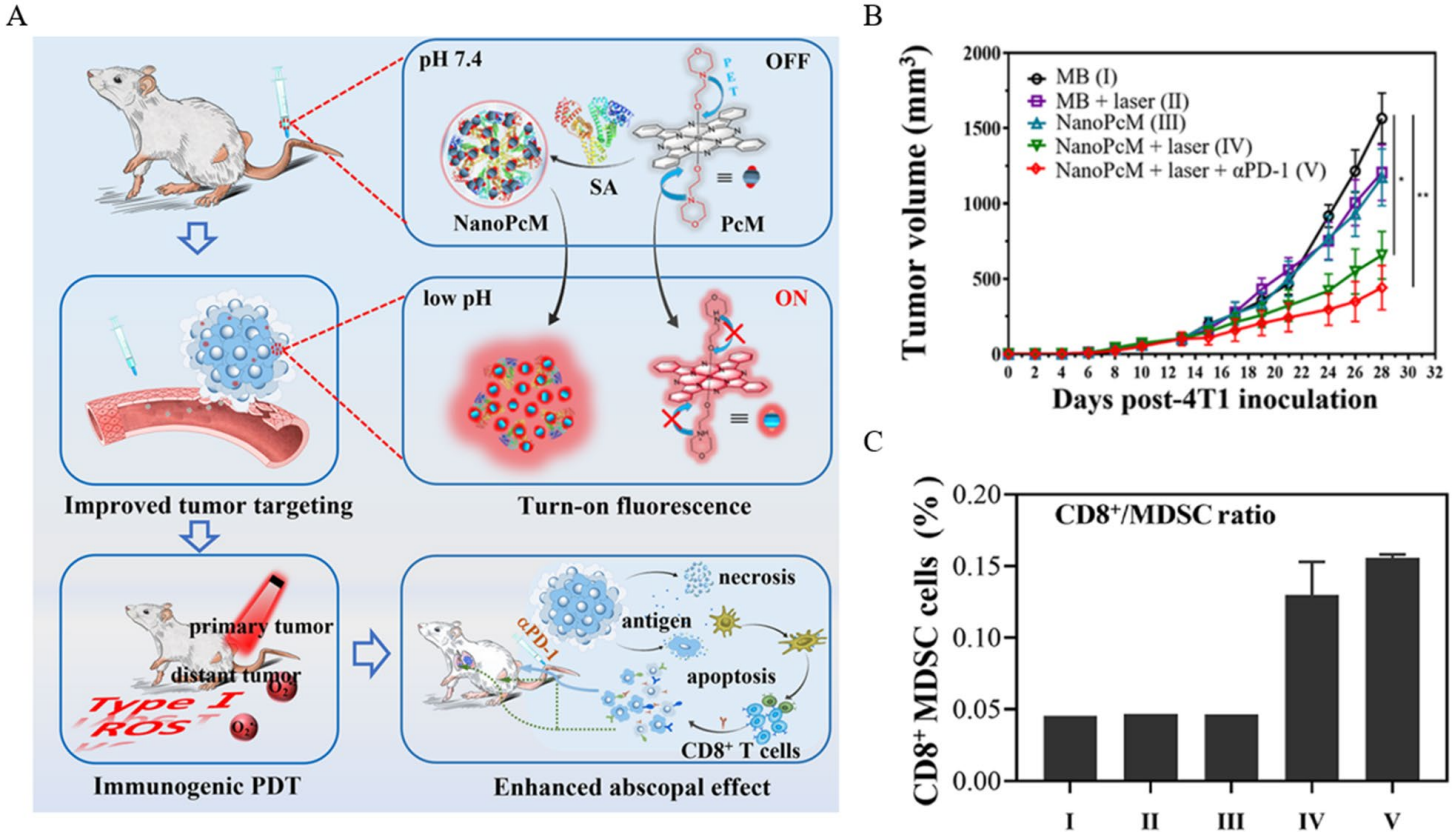
Anti-tumor efficacy of NanoPcM. **A** Schematic diagram of NanoPcM fluorescent signal "switch" and immunotherapy. **B** Tumor volume after receiving different treatment regimens. **C** CD8^+^T cell/MDSC ratio after receiving different treatment regimens. (Treatment groups: I: MB, II: MB + laser, III: NanoPcM, IV: NanoPcM + laser, V: NanoPcM + laser + αPD-1, *p < 0.05, **p < 0.01) Reprinted with permission from Ref. [164]. Copyright© 2022, copyright Wang et al.

The diagnosis and treatment of tumors have been two separate fields for a long time. Fortunately, with the promotion of bio-nanomaterials, the concept of integrated tumor diagnosis and treatment is gradually recognized and valued [162, 163]. Li et al. [164] constructed NanoPcM, a nanomedical platform for tumor fluorescence imaging and immunogenic photodynamic therapy, by self-assembling PcM (silica phthalocyanine) with albumin. The switch of fluorescence is controlled by pH, i.e., the low pH of TME induces the onset of fluorescence signal to achieve precise imaging of tumor sites. Importantly, the type I photo responsiveness demonstrated by NanoPcM ensures the effectiveness of its immunogenic PDT (Fig. 5A). After 4T1 tumor-bearing mice were treated with control and experimental groups, an increase in CD3^+^T and CD8^+^T cells and a decrease in the proportion of CD11b^+^ myeloid cells in tumor tissues after treatment with the NanoPcM + laser group could be observed. Considering that the infiltrated CD8^+^T cells in tumor tissues were of PD-1^+^TIM3^+^ phenotype, the team decided to block the PD-L1/PD-1 axis to enhance the anti-tumor efficacy. Unexpectedly, NanoPcM + laser + αPD-1 treatment effectively inhibited tumor growth (Fig. 5B). This process was accompanied by a significant increase in the CD8^+^T cell/MDSC ratio (Fig. 5C).

Since the eighteenth century, when Jenner inoculated boys with cowpox, conferring immunity to smallpox and describing the term "vaccine," more and more vaccines have been developed for the prevention and treatment of disease [165, 166]. Today, more than two centuries later, nanomaterial-based vaccines are being developed at an unprecedented rate. The advantages of nano vaccines include: (i) nanocarriers protect antigens from rapid degradation, thus ensuring the stability of the vaccine. (ii) Acting as adjuvant immune properties and promoting APC activation. (iii) The nano size facilitates lymphatic drainage [26, 167, 168]. However, attention should still be paid to technical difficulties such as improving antigens' loading rate and loading complex structures of antigens. Ma et al. [169] proposed a Nano-B5 nano vaccine based on protein self-assembly. The main structure of this nano is composed of subunits of bacterial ABs family toxin Bs with adjuvant properties as well as trimeric peptides. This strategy not only exhibits specific lymph node targeting in a tumor-bearing mouse model but also induces a robust immune response and an excellent biosafety profile. More clinically translatable, the experiments also used primates as models. The results show that the nano vaccine significantly activates the immune response due to the conventional vaccine. In the field of vaccine development, due to the low immunogenicity of viral structural proteins that self-assemble to form virus-like particles (VLP), a combination of adjuvants is required to represent the vaccine's function better. Sun et al. [170] constructed a VLP@Silica nano vaccine by exploiting the property that VLP and silica can self-assemble. The vaccine can be modularly tailored to the source of the virus. In a mouse model, VLP@Silica not only promoted DCs maturation but also diverted antigen to lymph nodes and effectively activated CD4^+^T and CD8^+^T. Although this study did not use tumor cells and tumor-bearing mice as experimental subjects, it provides an idea for constructing viral structural protein-derived nanomaterials for enhancing immunogenicity.

## Mesoporous nanomaterials

The effectiveness of antitumor therapy is frequently limited by poor water solubility and lack of specificity of drugs. Single treatment regimens may lead to incomplete treatment and recurrence of tumors, and the systemic administration of drugs may lead to more toxic side effects. In response to the above background, many scholars have proposed using mesoporous materials as carriers for tumor immunotherapy, which can effectively solve the problems of drug loading and combination application problems. It has received so much attention because of its high specific surface area, adjustable pore size, and modifiability. More importantly, it has the advantages of good biocompatibility and spontaneous degradation in vivo [171–173]. Mesoporous materials can be broadly classified into silicon-based and non-silicon-based, the same as they can be used for tumor immunization utilizing sonodynamic therapy (SDT), photothermal therapy (PTT) and photodynamic therapy (PDT), etc. This section also provides an overview of this classification.

### Mesoporous materials for SDT

**Fig. 6:**
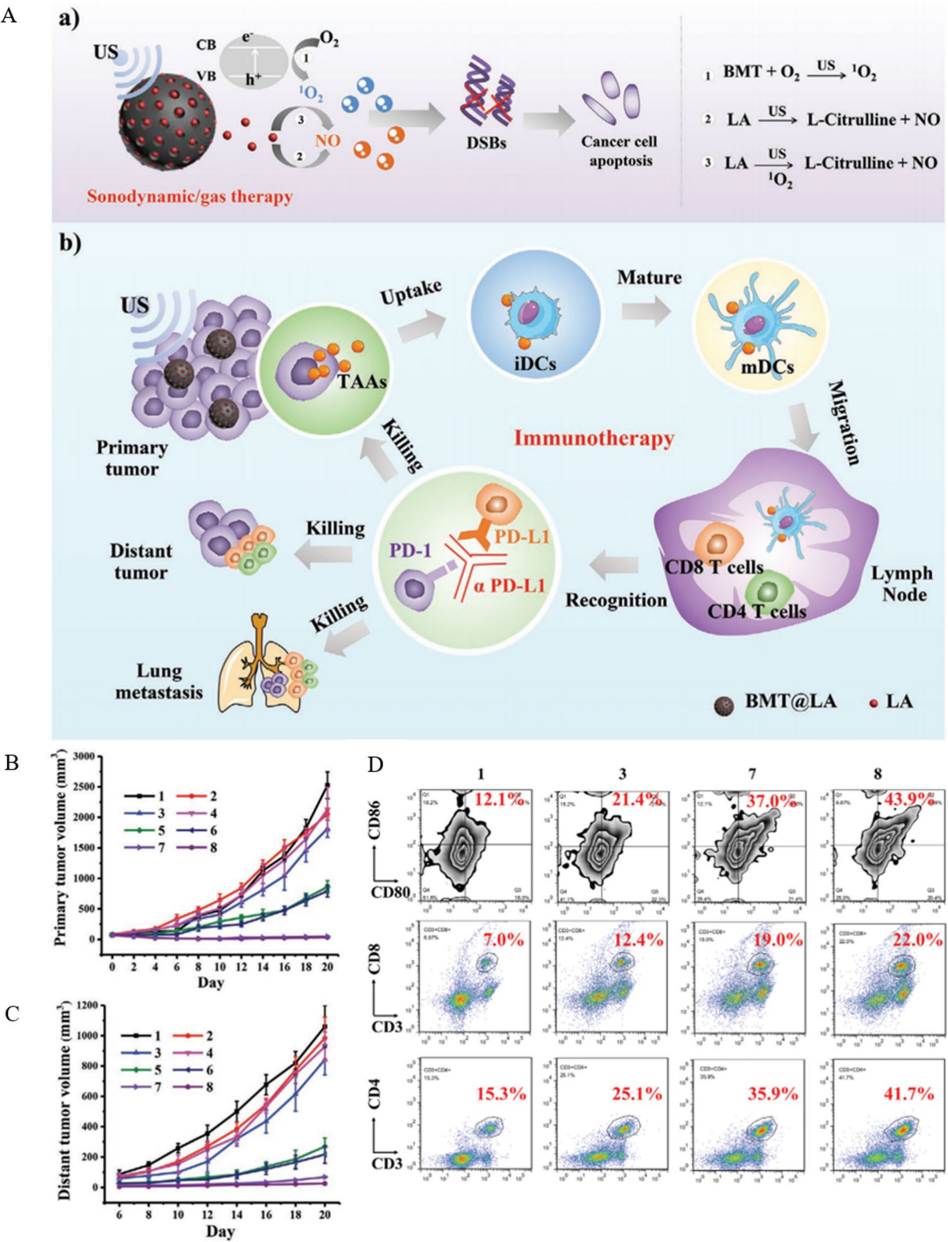
Anti-tumor efficacy of BMT@LA. **A** (**a**) Chemical formula of key components in BMT@LA when performing SDT/gas treatment. **b** Schematic diagram of BMT@LA inhibition of primary and distant tumors in immunotherapy. **B**, **C** Primary and distant tumor volumes in tumor-bearing mice after receiving different treatment regimens. **D** Flow cytometry analysis of the proportion of mature DCs (CD11^+^CD80^+^CD86^+^ as markers), CD8^+^T cells (CD3^+^CD8^+^ as markers), and CD4^+^T cells (CD3^+^CD4^+^ as markers) in the spleen after different treatment regimens (Treatment groups: 1: Ctl, 2: US, 3: α-PD-L1, 4: BMT@LA, 5: LA + US, 6: BMT + US, 7: BMT@LA + US, 8: BMT@LA + US + α-PD-L1) Reprinted with permission from Ref. [176]. Copyright© 2021, copyright Wang et al.

Titanium dioxide (TiO_2_) is a commonly used SDT acoustic sensitizer, and interestingly the color affects its performance, in which black TiO_2_ has a higher ability to produce singlet oxygen (^1^O_2_) than white TiO_2_. Considering the limited therapeutic effect of single SDT on tumors, SDT is commonly combined with other therapeutic options to fight tumors [174, 175]. Researchers have found that exogenous introduction of gases such as NO can achieve antitumor efficacy in the form of gas therapy. However, such delivery must be based on precise and controllable targeting. Otherwise, it can cause large side effects. Based on this, Lin et al. [176] synthesized BMT@LA spherical nanoparticles with a size of about 200 nm using black mesoporous titanium dioxide loaded with LA (l-arginine). LA was oxidized to l-citrulline and released NO under the action of US, which is a NO donor with good biosafety. Under the US, BMT@LA NCs can efficiently produce ^1^O_2_ on the one hand and release a large amount of NO on the other (Fig. 6A). Injecting BMT@LA into the tail vein of tumor-bearing mice to verify its efficacy, it was found that BMT@LA + US induced a potent anti-tumor immune response by promoting the maturation of DCs and increasing CD4^+^T and CD8^+^T cells. To extend the effect of synergistic treatment, the experiment combined α-PD-L1 for ICB, and the effect was validated using in situ and distant tumor models (Fig. 6B, C). The results showed that the regimen exhibited optimal suppression of both primary and distal tumors, which was attributed to the fact that BMT@LA + US + α-PD-L1 effectively enhanced the DCs maturation rate and successfully induced a potent immune response by increasing CD4^+^T cells and CD8^+^T cells (Fig. 6D). BMT@LA + US + α-PD-L1 also showed good performance in suppressing metastasis, which led to its designation as a "nano vaccine."

The immunosuppressive and hypoxic features of TME promote cancer malignancy, metastasis, and drug resistance [159, 177]. Therefore, reversing immunosuppression and alleviating hypoxia is an essential breakthrough in cancer treatment. Liu et al. [178] prepared HABT-C nanoparticles with mimetic enzyme activity using mesoporous hollow black TiO_2_ nanospheres as a carrier after loading gold nanoparticles and carbon dots. This enzymatic activity was demonstrated by: (i) the loading of carbon dots endowed the NPs with peroxidase activity, which decomposed the high level of H_2_O_2_ in tumor cells into H_2_O and O_2_ and solved the problem of tumor hypoxia. (ii) Au has glucose oxidase activity and converts glucose in tumor tissues into H_2_O_2_. (iii) Black TiO_2_ will catalyze the generation of ROS (·OH and ^1^O_2_) from H_2_O_2_, mimicking the peroxidase activity. In the subsequent experiments, the surface of HABT-C was modified with HA (hyaluronic acid) to enhance its dispersibility. Although the average hydrodynamic diameter was increased from 174 to 239 nm, the dispersibility was well improved. HA also played an essential role in the in vitro experiments because the hyaluronan receptor CD44 is a membrane protein highly expressed in tumors. The high affinity of HA and CD44 allows HABT-C@HA to be highly targeted to cancer cells. And HABT-C@HA was not toxic to normal cells HUVEC, on the contrary, manifested toxicity on tumor cells in a concentration-dependent manner. In vivo experiments, 4T1 tumor-bearing mice were administered by intravenous injection, and the tumor suppression efficiency of the HABT-C@HA + US group was 98% after 16 days. High-throughput sequencing of transcripts expressed (RNAseq) in tumor tissues and gene ontology (GO) enrichment analysis were performed. The results showed that genes related to tumorigenesis and progression, including OSM (Oncostatin M) and MMP-2, were significantly downregulated. Moreover, several immune-related pathways were significantly enriched, confirming that HABT-C@HA could achieve tumor treatment by regulating body immunity.

Similarly, to reverse the tumor immunosuppressive microenvironment, Zhang et al. [179] used mesoporous Prussian blue (MPB) with low molecular weight hyaluronic acid (LMWHA) modified on the surface to modulate the tumor microenvironment, using TAMs as a breakthrough. In vitro experiments, LMWHA-MPB uptake by M2 macrophages promoted their polarization toward M1 and showed the ability to inhibit tumor cell viability and migration. In an in vivo experiment on 4T1 tumor-bearing mice, LMWHA-MPB/HMME was obtained by loading LMWHA-MPB with HMME (hematoporphyrin monomethyl ether), an acoustic sensitizer used for its oxygen supply, to enhance its anti-tumor ability. The results showed that LMWHA-MPB/HMME + US effectively inhibited tumor growth and metastasis by reprogramming TME.

### Mesoporous materials for PTT

PTT is a form of therapy in which photothermal transducers (PTAs) convert absorbed photon energy into thermal energy and holds great promise in the biomedical field, especially in tumor therapy [180, 181]. According to the generation temperature, PTT can be divided into conventional photothermal therapy at ≥ 45 °C and MPTT (mild photothermal therapy) at < 45° [182, 183]. However, whether it is PTT or MPTT, the excellent biosafety and photothermal conversion efficiency of PTAs are necessary to achieve effective treatment. Compared with surgery and radiotherapy in traditional tumor treatment, PTT combines non-invasiveness and high temporal and spatial precision. PTT achieves tumor treatment while avoiding damage to normal tissues [184–186].

However, due to its limited effectiveness, tumors cannot be cured by a single PTT strategy alone and cannot prevent tumor metastasis and recurrence. Therefore, developing combination therapeutic strategies is particularly important, and immunotherapy based on ICB is a good "partner" for PTT [187, 188]. Ran et al. [189] used PEG to modify mesoporous silica shells with CuS nanoparticles at the core and loaded PFP (perfluoropentane) in the mesopores to obtain CuS@mSiO2-PFP-PEG (CPPs). CPPs can be integrated for tumor treatment under PA (photoacoustic) and US (ultrasound) guidance. CPPs are enriched at the tumor site by EPR and generate high temperature under NIR irradiation to directly kill tumor cells on the one hand and convert PFP into microbubbles on the other hand. The generation of microbubbles enhances the US imaging capability and in combination with the inherent PA imaging properties of CuS, achieves multimodal cancer therapy guidance. In vivo experiments, PTT released TAAs while killing tumor cells at a high temperature of about 53 °C. To enhance tumor treatment, the researchers added α-PD-1 to CPPs, and the results showed that the combination regimen effectively inhibited the growth of 4T1 primary and distant tumors. This was attributed to the combination strategy significantly elevating the infiltration of CD8^+^T cells in both primary and distant tumors and significantly increasing the levels of immune-related factors, including TNF-α, IFN-γ, IL-4, IL-6 and IL-12. The same was achieved by MSNs (mesoporous silica nanoparticles) for PTT, and Huang et al. [190] synthesized CD@MSN using a co-assembly strategy to introduce carbon nanodots (CDs) into the MSN framework. Compared with conventional MSNs, CD@MSN has better degradability. The results showed that CD@MSNs were enriched in tumor tissues through the EPR when NIR irradiation was given to fragment them. These TAAs-carrying nanofragments effectively inhibited the growth of in situ and distant tumors by activating the body's immune cells. Whether adjusting the nanosize, surface modification to enhance their delivery efficiency or modification of their structural composition, it is sufficient to demonstrate the great clinical value of MSNs in tumor immunotherapy.

The phenomenon of low pH in solid tumors is often attributed to lactate accumulation due to the reprogramming of metabolic pathways in hypoxic tumors. This lactate-driven "Warburg effect" directly affects immune regulation, including promoting M2 polarization and suppressing the toxicity and activity of T lymphocytes and NK cells [191–193]. Despite the negative regulation of lactate in immunotherapy, strategies that target lactate metabolism are thought to break the bottleneck of cancer immunotherapy. Researchers have tried to achieve the above problem by introducing LOX (lactate oxidase) that can consume lactate, but the process of LOX catalyzing lactate to pyruvate requires oxygen consumption [194]. On the one hand, the hypoxic environment of tissues limits the action of LOX, and on the other hand, the oxygen-consuming property aggravates the lactate accumulation in tumor tissues [195, 196]. To break the deadly cycle of LOX-catalyzed lactate, Dong et al. [197] loaded Cu^2+^ and LOX onto mPDA (mesoporous polydopamine nanoparticles) by ligand and electrostatic adsorption. They then modified the mesoporous surface with PEG to obtain the end product, mCuLP. Thanks to the modification by PEG, mCuLP releases LOX in a tumor-specific acidic microenvironment at a fixed point. LOX consumes lactate to promote M1 polarization and restore T-cell function. This process has an adequate supply of oxygen because the CAT (catalase) activity of Cu^2+^ in mCuLP catalyzes the conversion of H_2_O_2_ into O_2_. Meanwhile, tumor cells' mild PTT irradiation (44 °C) exposes DAMPs, stimulates DC maturation, and promotes CTL activation and infiltration. In an in vivo experiment using 4T1 tumor-bearing mice as a model, the mCuLP + NIR group exhibited significantly better tumor suppression than the PBS, LOX, mCuP + NIR, and mCuLP groups. The M1/M2 macrophage ratio, lymph node mature DCs and splenocyte CD3^+^CD8^+^CTL in the tumors of the mCuLP + NIR group were 2.72, 2.94 and 1.28 times higher than those of the PBS control group, respectively. It indicates that mCuLP + NIR effectively reversed the tumor immunosuppressive microenvironment by regulating lactate metabolism and initiated a potent anti-tumor systemic immune response.

Considering the superior performance of mPDA, Cai et al. [198] loaded TLR7 agonist R837 was loaded into it and surface modification was performed using PVP to obtain PVP-MPDA@R837 NPs. This strategy delivered R837 well into the lymph nodes with its unique nanoscale and ensured its long-lasting retention, allowing it to activate DCs effectively. The activated DCs present TAAs released by PTT treatment to T cells, inducing cytotoxic T lymphocytes to kill tumors.

### Mesoporous materials for PDT

As a non-invasive treatment, PDT uses a photosensitizer to deliver energy to the surrounding oxygen under laser irradiation at a specific wavelength to generate cytotoxic singlet oxygen to kill tumor cells [199, 200]. PDT has been incorporated into various tumor treatment regimens due to its controllability and selectivity in target organs and degree of damage. In addition, PDT also stimulates the body's anti-tumor immune response by inducing ICD to kill tumor cells [201, 202].

In tumor immunity, the recruitment of DCs and their activation are crucial for the presentation of TAAs to activate T cell function. ROS generated by PDT was found to play a key role in DC recruitment. In the premise of this study, Dong et al. [203] loaded Ce-6 into bullet-shaped Janus magnetic mesoporous organosilica nanoparticles (M-MONs). And the CM@M-MON@Ce6 was obtained by using the homologous targeting ability of breast cancer cell membrane wrapping in the outermost layer. Unlike conventional round spherical mesoporous silica nanoparticles, this mesoporous framework structure containing disulfide bonds is characterized by redox/pH dual responsiveness, ensuring the system's targeted release at the tumor site. In vitro assays, CM@M-MON@Ce6 efficiently induced CRT exposure in MCF-7 cells under the laser and ACMF stimulation and HMGB1 release, critical indicators of ICD. In vivo experiments, the circulating half-life of NPs was 4.7-fold higher than without cell membrane modification. It was more efficiently enriched in tumor tissues due to tumor cell membrane modification. The CM@M-M-MON@Ce6 + laser + ACMF regimen was used to treat the tumor-bearing mice. The results showed that the tumor suppression rate of this regimen was significantly higher than the rest of the treatment groups and effectively reduced the number of metastatic tumors. These promising results were attributed to a significant increase in the maturation level of DCs in primed LNs, increased recruitment of CTL in tumor tissues, decreased Treg, and other immune factors. To enhance the efficacy of CM@M-MON@Ce6 + laser + ACMF immunotherapy, the investigators included α-CTLA-4 in the treatment regimen, ultimately achieving primary tumor eradication and metastatic tumor suppression.

**Fig. 7:**
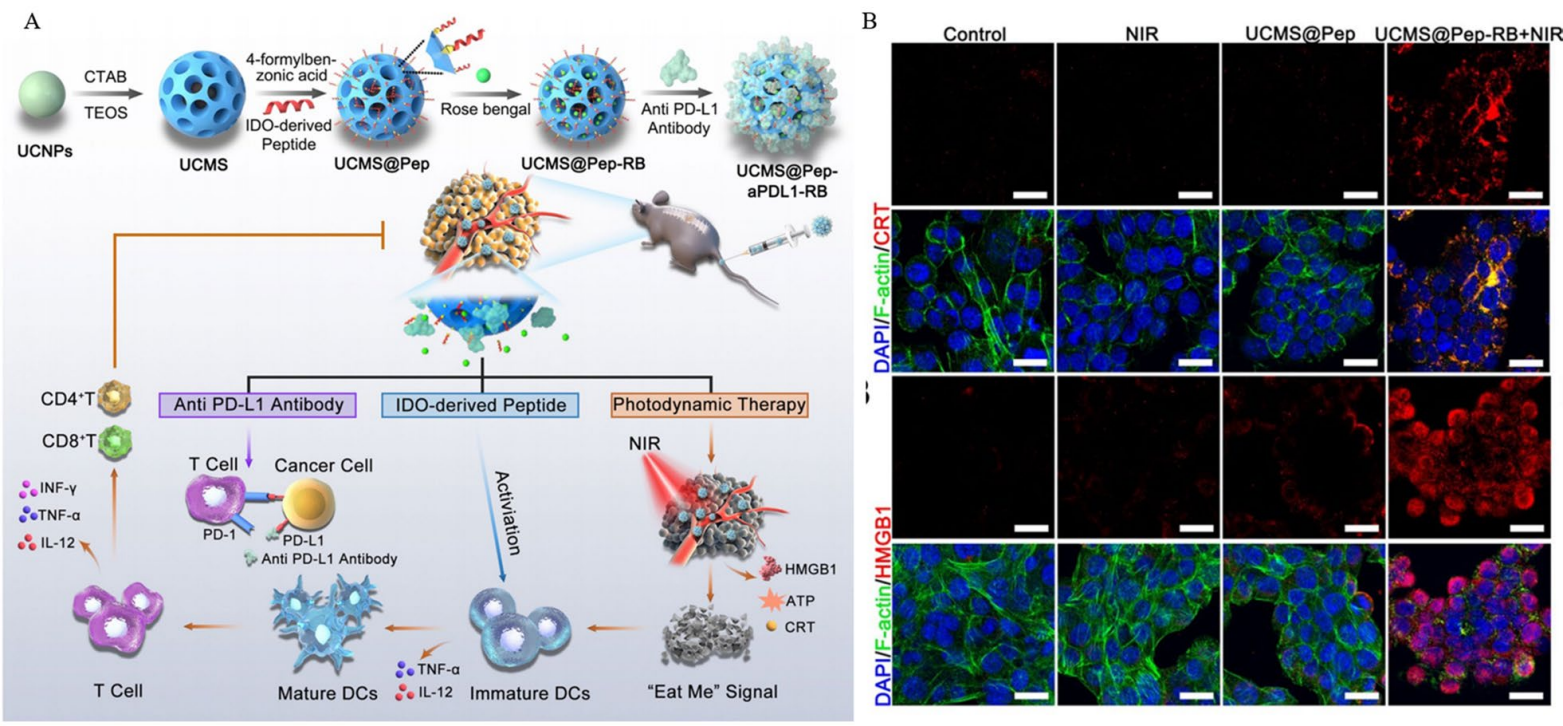
Anti-tumor efficacy of UCMS@Pep-αPDL1. **A** Schematic diagram of UCMS@Pep-αPDL1 synthesis pathway based on mesoporous nanomaterials and its use in tumor immunotherapy. **B** CLSM observation of immunofluorescence images of CRT and HMGB1 in Lewis murine lung carcinoma cells after receiving different treatment regimens (Scale bar = 25 μm). Reprinted with permission from Ref. [206]. Copyright© 2021, copyright Wang et al.

Bone metastasis caused by lung cancer seriously affects patients' quality of life and survival. The existing treatments are based on surgery, chemotherapy and radiotherapy, but the treatment effect is unsatisfactory [204, 205]. Although an increasing number of immunotherapy regimens represented by PD-1/PD-L1 inhibitors are being incorporated into the treatment of advanced and metastatic tumors, their clinical effectiveness remains limited due to a firm TIME. To improve this therapeutic dilemma, Dong et al. [206] constructed an immune response-based all-encompassing multi-targeted mesoporous nanoformulation UCMS@Pep-αPDL1. The brief preparation process was that hexagonal UCNPs (upconversion nanoparticles) with a diameter of about 40 nm were coated with dense SiO_2_ and macroporous mesoporous silicon on the surface to obtain UCMS, and finally loaded with photosensitizer RB (Rose Bengal), IDO-derived peptide vaccine AL-9, and PD-L1 inhibitor (Fig. 7A). In vitro experiments, UCMS@Pep-αPDL1 + NIR not only effectively induced tumor cell apoptosis but also produced a large amount of ROS, accompanied by the release of ICD markers ATP, HMGB1 and CRT (Fig. 7B), and promoted the maturation of DCs. As a model, in vivo metabolism and biosafety experiments were performed in mice with spinal cord metastases. The circulating half-life was measured to be 0.96 h after intravenous injection of Cy7-labeled NPs. The signal intensity at the tumor site peaked at 12 h after injection and was still observed 24 h later. Blood biochemistry and H&E staining of major organs showed that UCMS@Pep-αPDL1 did not cause significant changes in detectable indicators or liver and kidney toxicity, indicating its good biocompatibility. In the evaluation of in vivo tumor treatment effect, the UCMS@Pep-αPDLI-RB + NIR laser group showed the smallest tumor size and significantly increased apoptosis. Immunometric assays revealed that UCMS@Pep-αPDLI-RB induced DC maturation and infiltration of T cells. Its ability to induce DCs maturation was 1.93 times higher than that of the PBS group, and the percentages of CD4^+^T and CD8^+^T cells were increased by 2.75 and 2.84 times, respectively. Ultimately, the UCMS@Pep-αPDL1 strategy stimulated local and systemic anti-tumor immunity through PDT-induced ICD in combination with ICB, reducing the progression of bone metastases.

Similarly, for diffuse and metastatic cancers, Moon et al. [207] proposed loading neoantigen, CpG and Ce6 onto biodegradable mesoporous silica nanoparticles (bMSNs) to obtain bMSN (CpG/Ce6)-Neoantigen with a size of about 80 nm. For more specific diagnostic purposes, the team loaded the radioisotope ^64^Cu on NPs for PET (positron emission tomography). The results showed that bMSN (CpG/Ce6)-neoantigen effectively recruited DCs and activated CD8^+^T after combined NIR irradiation. This strategy achieved good therapeutic results in mice with primary and distant metastatic tumor-bearing mice. This mesoporous material-based nanoplatform with therapeutic integration provides ideas for treating advanced tumors.

This section reviewed mesoporous nanomaterials for SDT, PDT, and PTT, which are both silicon-based and non-silicon-based NPs consisting of, for example, polydopamine. Similarly, all these solutions are used for non-invasive tumor immunotherapy by external factors such as laser or ultrasound. The high loading properties of mesoporous materials allow them to deliver large amounts of acoustic sensitizers, PTAs and PS and to achieve efficient treatment under specific excitation. Of course, there are a number of differences among them, including: (i) their excitation methods are different, as PDT and PTT are mesoporous materials functioning under laser irradiation, while SDT excitation energy comes from ultrasound. (ii) The different ways of achieving tumor treatment, although they both ultimately point to immunotherapy, the process is dominated by ROS generation for PDT, while SDT and PTT are dominated by raising the temperature of the tumor site. (iii) The difference in response modality makes them different in the design of mesoporous materials, for example, mesoporous materials for ROS-responsive dissociation can directly respond to ROS generated by PDT, while in SDT and PTT the dissociation purpose can only be achieved by responding to ROS highly expressed by cells. Notably, these mesoporous material-based therapeutic modalities are often not monolithic; they are more often in the form of combination therapies aimed at maximizing the effect of immunotherapy.

## Metallic nanomaterials

Metal ions and related proteins are required for almost all life processes, such as enzyme cofactors, signal transduction, structural support, and energy transfer. In addition, metals also play an essential role in immune regulation, such as immune activation and host defense [208, 209]. It cannot be ignored that metal-based drugs, including cisplatin and aluminum adjuvants, are widely used in clinically treating tumors [210, 211]. Researchers have continued to explore the relationship between metallic nanomaterials and tumor immunotherapy and have achieved excellent results. Given the potential of metal nanomaterials, this section will review the applications of metal atoms, metal ions and particles based on the metal in tumor immunotherapy.

### Metal atoms-based nanomaterials

Enzymes are highly specific and catalytically efficient and are essential for regulating biological processes, including tumors. Therefore, introducing enzymes through exogenous sources to regulate biological functions has a promising future. Due to the dilemma that enzymes of natural origin are prone to deactivation and denaturation and difficult to obtain, nanoenzymes with enzymatic functions have been proposed. Thanks to their good catalytic activity, stability and relatively simple synthesis process, nanoenzymes have become one of the most promising bio-nanomaterials [212–214]. Among them, due to the maximum utilization of metal atoms, single-atom nanoenzymes (SANs) with vigorous enzyme-like activity have gained widespread attention in biomedicine, especially in tumor therapy. Based on the unique catalytic properties of SAN, Wang et al. [215] prepared Pd single-atom nanoenzymes (DA-CQD@Pd SAN) using by polyphenol carbon quantum dot template method. And the catalytic property of SAN was used to obtain injectable hydrogel DA-CQD@Pd@CpG ODN with an adhesion effect composed of DA-CQD@Pd SAN and immune adjuvant CpG ODN for tumor immunotherapy. Compared with the more significant toxicity of systemic administration, local injection protects CpG ODN from degradation and allows continuous delivery to the tumor site. In vitro experiments have shown that SAN possesses peroxidase-like activity by catalyzing the production of ROS from H_2_O_2_ to kill tumor cells. In vivo experiments using CT26 tumor-bearing mice as a model, DA-CQD@Pd@CpG ODN effectively suppressed tumor growth and improved survival by promoting CD4^+^T cells, CD8^+^T cells, and inhibiting Treg and M2 macrophages. To extend the effect of immunotherapy, the efficacy was validated using a bilateral CT26 tumor model for the control group and each experimental group, including DA-CQD@Pd@CpG ODN + α-PD-L1. The results showed that the combination regimen had the most significant distal tumor suppression effect and a 30-day survival rate of 80% in mice.

Despite the high catalytic activity of peroxidase-like SAzyme, the limited H_2_O_2_ concentration and the TIME limit its antitumor efficacy [216]. Fan et al. [217] used agarose hydrogels loaded with Pd–C SAzymes and camptothecin to obtain LOA with oxidative stress amplification efficacy under light control. The PTT property of Pd–C Sazyme converts NIR into thermal energy to promote agarose degradation and release of camptothecin. Camptothecin ensures efficient catalysis of SAzymes by activating nicotinamide adenine dinucleotide phosphate oxidase and increasing H_2_O_2_ levels in tumors. The ROS produced by Pd–C Sazyme and the TAAs released by the tumor under the action of PTT synergistically promote the recruitment of DCs and increase the CD8^+^T cells, ultimately activating the body's immune response. This combination of multiple regimens of antitumor immunity reveals the great clinical potential of Sazyme.

**Fig. 8:**
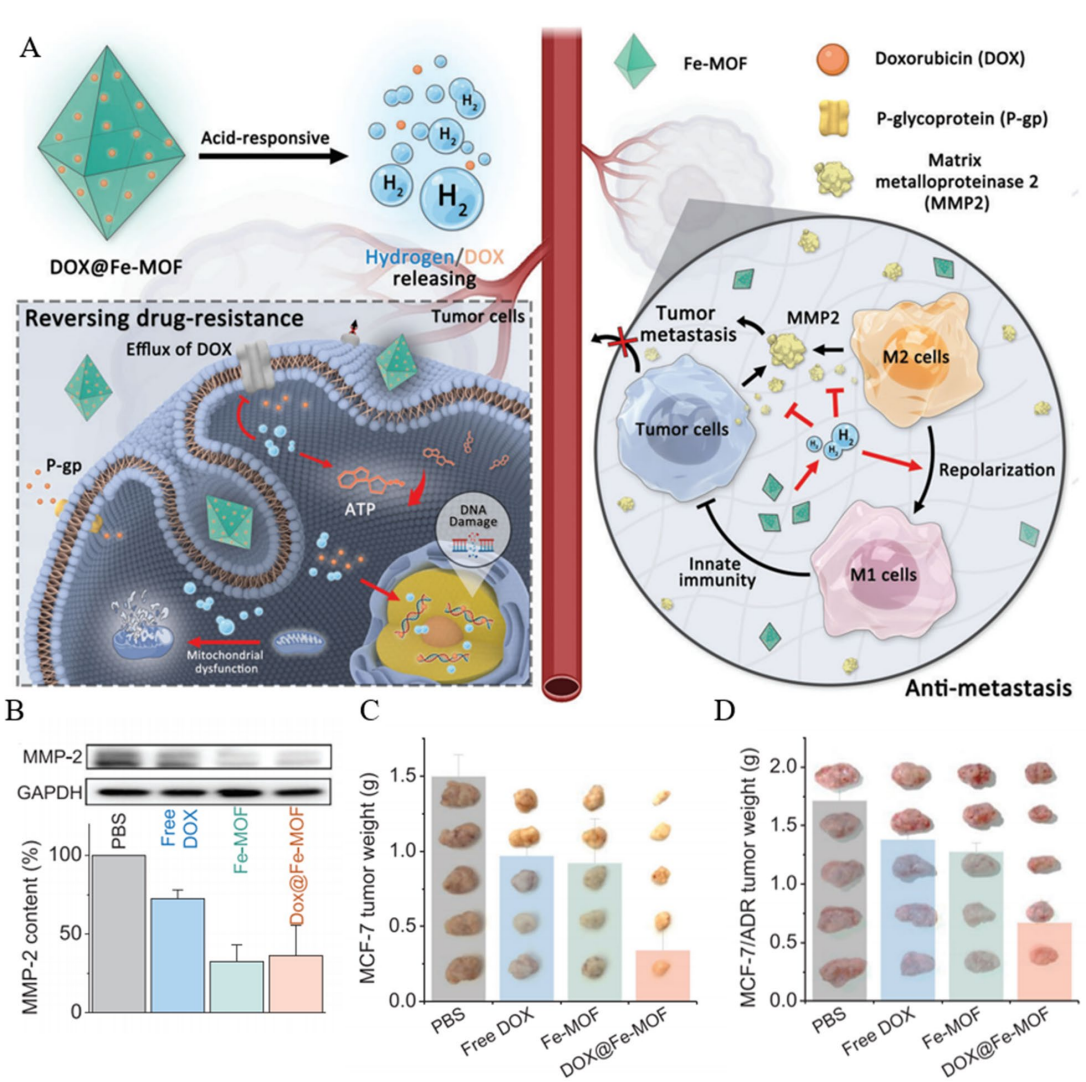
Anti-tumor efficacy of DOX@Fe-MOF. **A** Schematic diagram of DOX@Fe-MOF to overcome cancer drug resistance and metastasis through H_2_ production in acidic environment **B** Western blot detection and quantification of MMP-2 expression in tumors after different treatment regimens. **C**, **D** Tumor weights in MCF-7 and MCF-7/ADR (ADR-resistant) tumor-bearing mice after receiving different treatments. Reprinted with permission from Ref. [220]. Copyright© 2022, copyright Yao et al.

MOFs (metal–organic frameworks) are a class of porous coordination polymers composed of metal ions or metal atoms/clusters attached to organic ligands through coordination bonds [218, 219]. They are widely used in drug delivery and imaging because of their powerful drug loading capability, modifiability, and controlled release. He et al. [220] prepared an acid-responsive and H_2_ supply porphyrin-iron metal–organic backbone (Fe-MOF) using the coordination of porphyrins with zero-valent iron atoms. It synthesized DOX@Fe-MOF nanoparticles by loading the chemotherapeutic drug DOX (Fig. 8A). In the tumor-specific acidic environment, DOX@Fe-MOF undergoes degradation and will produce H_2_ and release the loaded DOX. The released H_2_ downregulates the expression of MMP-2 (Fig. 8B), a tumor metastasis-associated protein, by promoting the polarization of M1 and downregulates the expression of P-gp, a protein with drug efflux pump function, thus ensuring the effective drug concentration in tumor cells. Ultimately, DOX@Fe-MOF can effectively inhibit tumor growth and metastasis in vivo experiments using MCF-7, MCF-7/ADR (Fig. 8C, D) and 4T1 tumor-bearing mice as models, and this H_2_-based treatment regimen can effectively restore the susceptibility of drug-resistant tumors. Given the excellent performance of metal-atom-based MOF materials in reversing tumor resistance and fighting tumor metastasis, we believe that such drugs have a high potential for clinical translation.

### Metal particle-based nanomaterials

MHT (magnetic heat treatment) is a new type of tumor treatment solution that utilizes the heat generated by magnetic nanoparticles under the action of AMF (alternating magnetic field) to kill tumors [221, 222]. Thanks to the substantial penetration depth of the magnetic field, it has good clinical translation significance for deep tumor treatment. And MHT is often bionically focused on the tumor site by intratumoral injection, and this minimally invasive injection scheme avoids the toxic side effects of systemic drug delivery. However, the wide application of MHT is still limited by the following points: (i) The commonly used MHT agent SPIONs (superparamagnetic iron oxide nanoparticles), despite its high biosafety, still needs to be improved due to the low saturation magnetization intensity and the induced tumor treatment effect. (ii) The targeted heating approach cannot control the spread of metastatic tumors [223–225].

Therefore, Liu et al. [226] proposed a PEG-functionalized iron nanoparticles (FeNPs) with high magnetic saturation intensity for MHT and combined with an immune adjuvant and ICB. FeNP-PEG has good dispersion and stability in an aqueous solution and allows its storage in lyophilized form. Considering that systemic administration of NPs may cause extreme factor storms, the team used the local injection of FeNPs + immune adjuvant PR (PLGA-R837) in combination with AMF to treat tumor-bearing mice. Analysis of treated tumors revealed significant upregulation of the ICD marker HMGB1 and efficient induction of CD8^+^T cell conversion to effector memory T cells in the spleen. This suggests that the combination strategy has tumor vaccine efficacy for ICD induction. To test whether the FeNP-PEG-based regimen could achieve metastases treatment, the team added α-CTLA4 to the treatment and designed a FeNP + PR + AMF + α-CTLA4 group. The results showed that only the FeNP + PR + AMF + α-CTLA4 group significantly inhibited primary and distant tumors compared to other treatment groups. This was accompanied by a significant increase in serum cytokines such as TNF-α and IFN-γ. This strategy ensured the survival of all mice during the 60-day observation period. In conclusion, this metal ion-based MHT combined with immunotherapy extends the therapeutic options for patients with advanced tumors, especially those with distant metastases.

The conversion of immunosuppressive "cold tumors" to immunosensitive "hot tumors" is a hot topic of research to expand the therapeutic effect of ICB at this stage. PTT-induced ICD is a good promoter of this conversion, and PTT can also down-regulate the expression level of PD-L1. Therefore, PTT is considered a good partner for ICB, but there are still some shortcomings for PD-1/PD-L1 blockade, primarily administered in the form of antibodies at this stage [227]. CRISPR/Cas9 editing technology, which has been hotly researched in recent years, has been proposed to modulate immune checkpoints to achieve better therapeutic effects [228–230]. Ping et al. [231] developed ANP/HSP-Cas9 nanoparticles by combining gold nanorod (ARs)-based PTT with CRISPR/Cas9-mediated PD-L1 downregulation. This is because laser irradiated ARs provide the optimal temperature of 42 °C required for Cas9 transcriptional activation, ensuring efficient editing of PD-Ll in tumor cells. In vitro experiments confirmed that the temperature increase mediated by laser irradiation assisted the CRISPR editing system and effectively down-regulated the PD-L1 expression. This process was accompanied by more CRT exposure and HMGB1 release and promoted DCs maturation. In the treatment experiment for B16-F10 tumor-bearing mice, tumors were significantly suppressed, and survival time was effectively prolonged after ANP/ HSP-Cas9 + NIR-II laser irradiation. This is because the combined strategy promoted the maturation of DCs in vivo, effectively boosted the number of CD8^+^T and CD4^+^T cells at the tumor site and in the immune organ spleen, and enhanced the intratumoral infiltration of immune cells. At the same time, it was found that the proportion of immunosuppressive cells, such as Treg and M2 cells, associated with the dominant "cold tumor" was significantly reduced [232, 233]. The above immune activation results also ensure therapeutic efficacy in metastatic tumor models, providing a long-term immune memory effect and effectively preventing tumor metastasis. In conclusion, this therapeutic modality based on a good photothermal conversion effect of metals amplifies the effect of CRISPR/Cas9 and offers the possibility of ICB-based immunotherapy.

### Metal ions-based nanomaterials

**Fig. 9:**
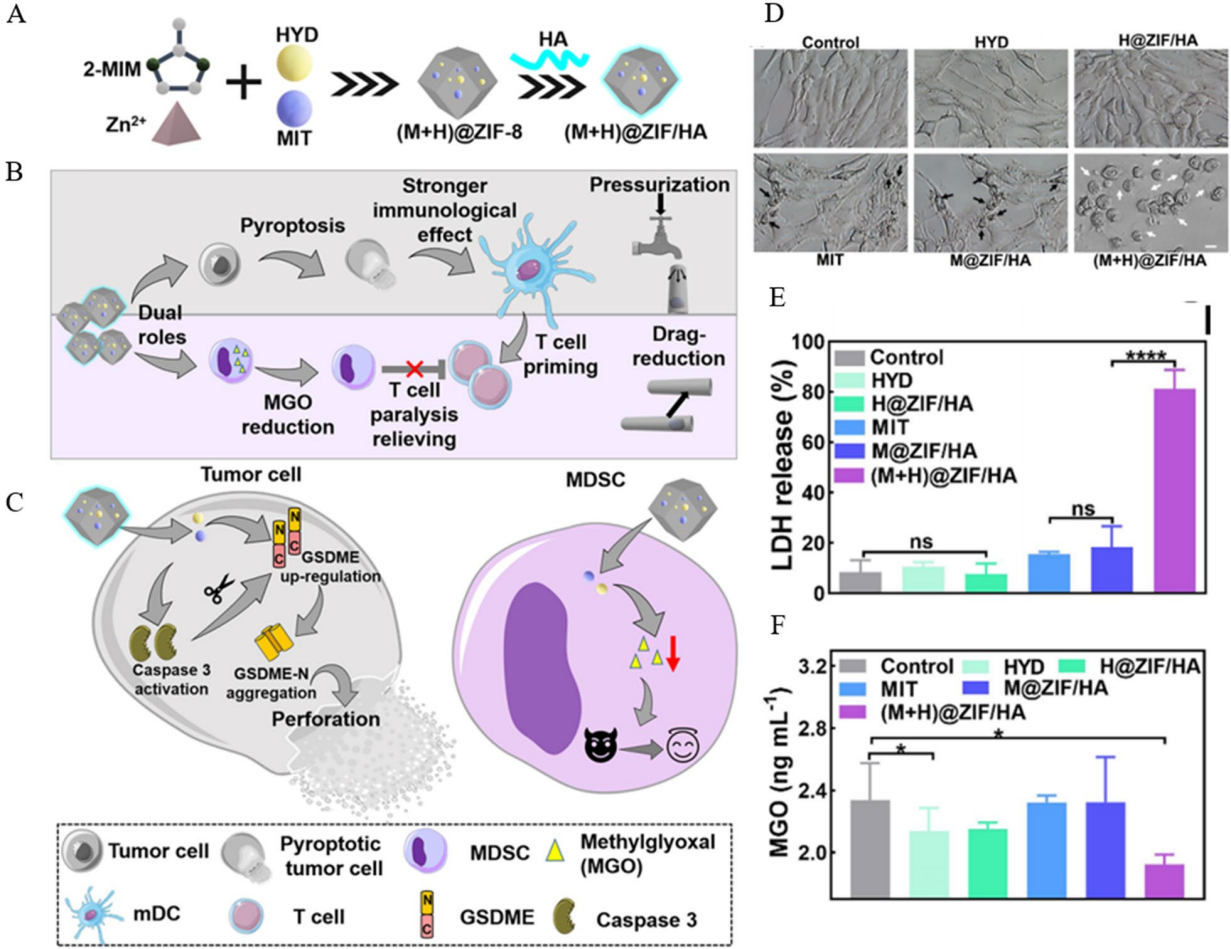
Anti-tumor efficacy of (M + H)@ZIF/HA. **A** Schematic diagram of (M + H)@ZIF/HA synthesis. **B** Schematic diagram of (M + H)@ZIF/HA for tumor immunotherapy through multiple pathways including induction of pyroptosis. **C** Schematic diagram of (M + H)@ZIF/HA in inducing tumor cell pyroptosis and taming MDSC. **D** Observation of 4T1 cell morphology after different treatments (apoptotic cells: black arrows; pyroptotic cells: white arrows. Scale bar = 10 μm). **E** Leakage of intracellular content of the LDH after diverse treatments. (F) MGO concentration in MDSCs after different treatments (*p < 0.05, ****p < 0.0001). Reprinted with permission from Ref. [238]. Copyright© 2021, copyright Zhou et al.

Recently, novel regulated cell death, including pyroptosis, ferroptosis, anoikis, and cuproptosis, have been proposed for tumor therapy [234–236]. Among them, pyroptosis, which is mainly mediated by the GSDM family, has gained widespread attention. Unlike conventional chemotherapy regimens that induce apoptosis, the occurrence of pyroptosis is accompanied by more intense immunogenicity [237]. However, this mediated tumor immunotherapy is limited by myeloid-derived suppressor cells (MDSCs). Based on this study, Luan et al. [238] proposed a nanoparticle that could disrupt MDSC immunosuppression and induce cellular pyroptosis with high efficiency. Zn^2+^-based pH-responsive ZIF-8 (zeolite imidazolium framework-8) was used as a carrier for the nanomaterials, and (M + H)@ZIF was obtained by loading the drug MIT (mitoxantrone) and the DNA demethylating agent HYD (Hydralazine) (Fig. 9A–C). Finally, (M + H)@ZIF with a hydrated particle size of about 176.3 nm was obtained by modifying (M + H)@ZIF/HA using negatively charged HA. In the morphological observation experiments, the laser (M + H)@ZIF/HA-treated cells showed a pyroptosis morphology with cell expansion and large bubbles compared to the morphology of apoptosis induced by MIT and M@ZIF/HA (Fig. 9D). And the content of LDH (lactate dehydrogenase) and the expression of the GSDME-N structural domain was significantly elevated in the cells of the (M + H)@ZIF/HA group (Fig. 9E). The MDSCs-specific metabolite MGO causes immunosuppression by affecting the metabolism and function of T cells. The team found a significant decrease in MGO concentration in MDSCs after (M + H)@ZIF/HA treatment in the experiments, which was an essential intervention for relieving the suppression of T cells by MDSCs (Fig. 9F). In vivo experiments, thanks to the HA modification, enhanced drug aggregation at the tumor site, ensuring that the drug achieves specific action against the target organ without damaging the remaining organs. In the treatment of tumor-bearing mice, (M + H)@ZIF/HA inhibited tumor growth and metastasis well compared to the control group and the rest of the treatment group and demonstrated almost negligible systemic toxicity. These impressive results were attributed to (M + H)@ZIF/HA's conversion of non-inflammatory apoptosis to inflammatory pyroptosis, which promoted DCs maturation while enhancing CTLs and helper T cells infiltration at tumor sites. Significantly, downregulation reduced the infiltration of key "cold tumor" cells, MDSCs and Treg. The icing on the cake was a significant increase in the proportion or number of CTLs, helper T cells and memory T cells in the immune organ, which further supported that (M + H)@ZIF/HA induced potent and diverse anti-tumor immunity.

In another study based on novel metal-induced cell death in tumor immunotherapy, Xu et al. [239] proposed using a gel system based on gold nanorods (AuNRs) and iron oxide nanoparticles (ions) to achieve perfusion therapy for bladder cancer. The Gel containing glucuronide ensures that AuNRs&IONs@Gel reach the lesion site by effectively adhering to bladder cancer collagen. This mode of administration by bladder perfusion effectively avoids the capture of Kupfer cells during systemic administration. AuNRs under NIR irradiation induced tumor cell death via PTT. Meanwhile, the high local concentration of ferrous ions polarized M2 to M1 and reversed the TIME and induced ferroptosis of tumor cells. This metal particle and particle-based non-invasive drug delivery halted bladder cancer progression in multiple ways including PTT, ferroptosis, and tumor immunotherapy, which is of extraordinary significance for its treatment.

STING (stimulator of interferon genes) has a solid theoretical foundation in tumor immunotherapy as a bridge between intrinsic and adaptive immunity. However, the poor stability of STING agonists and limited cell permeability have limited its promotion in the clinical setting. With the emergence of tumor immunotherapy, researchers have discovered that metal ions play an important role in immunomodulation and have proposed the concept of "metal immunotherapy" [240, 241]. Moon et al. [242] found that Mn^2+^ could significantly enhance the activity of STING agonist IFN-I through screening and designed a self-assembled nanoparticle CMP (CDN-Mn^2+^ particle) based on Mn^2+^ and CDN STING agonist, and prepared CMP_CDA_ by modification. CMP_CDA_. In vitro experiments, CMP_CDA_ was significantly taken up by BMDCs and exhibited a strong ability to promote IFN-β secretion by BMDCs. In vivo, intratumoral injection of CMP_CDA_ inhibited tumor growth by inducing elevated levels of IFN-β, TNF-a, CXCL10 and CCL5, and CD8^+^T cell responses. At the same time, CMP_CDA_ intratumoral NK cell infiltration and tumor-draining lymph nodes were observed. To address STING agonists' limitations for treating metastatic tumors, the team also added systemic dosing to the treatment regimen in animal studies. As with intratumoral administration, intravenous administration also induced a potent immune response and broke through the limitations of STING agonist use.

## Hydrogel nanomaterials

While researchers use nanomaterials as drug delivery vehicles for treating diseases, they pay equal attention to their biosafety. Hydrogels are widely used in the biomedical field because of their superior biocompatibility and are considered systems that meet these criteria. At the same time, the hydrogel is a polymeric network structure with high drug loading efficiency [243, 244]. Researchers have determined the release of loaded drugs by modification of hydrogels and modulation of the mesh size, which has led to their frequent design for tumor immunotherapy. Because of the great promise of hydrogels in tumor immunotherapy, their classification is diverse. Some classify them according to the difference in the mode of drug delivery or the way they are subjected to the external response [245]. However, considering the many recent advances in the rapid development of hydrogels, we will classify them in this section by the structure of their constituent end products, including scaffolds, sprays, and microneedles. Also, we will summarize the application of oral hydrogels in tumor immunotherapy as distinguished from traditional delivery methods such as intravenous and in situ injections.

### Hydrogel spray

As an essential member of the immune defense, macrophages can phagocytose "non-self" substances and present them to T cells. Usually, tumors are "non-self" substances, but cunning tumor cells avoid phagocytosis by expressing CD47 "don't eat me" signaling proteins on the cell membrane surface that bind to SIRPα on the macrophage membrane. Thus, blocking the CD47/SIRPα signaling pathway was evaluated as a viable antitumor regimen to avoid unnecessary systemic toxicity associated with systemic drug delivery. At the same time, the researchers noted that the microenvironment of the wound after tumor resection is acidic, and such an environment promotes an increase in M2 macrophages, which is detrimental to antitumor therapy. Therefore, Gu et al. [246] prepared to spray A component αCD47@CaCO_3_ and B component containing thrombin by mixing CD47 antibody and fibrin gel loaded with calcium carbonate nanoparticles. The interaction of fibrinogen and thrombin ensured wound healing while also assuming the function of a drug reservoir by spraying the A and B components separately into the post-tumor wound. Among them, CaCO_3_ and αCD47 reversed the acidic microenvironment-induced M1 polarization and promoted macrophage phagocytosis by blocking the CD47/SIRPα pathway. In vivo experiments, the researchers found that the regimen not only effectively modulated the phenotype of macrophages but also downregulated the number of immunosuppressive marker cells MDSCs and Treg and reduced the expression of HIF1-α (hypoxia-inducible factor 1-α) [247, 248]. Ultimately, the gel spray protocol inhibited tumor recurrence by activating the body's immune system and effectively prolonged the survival time of mice.

**Fig. 10:**
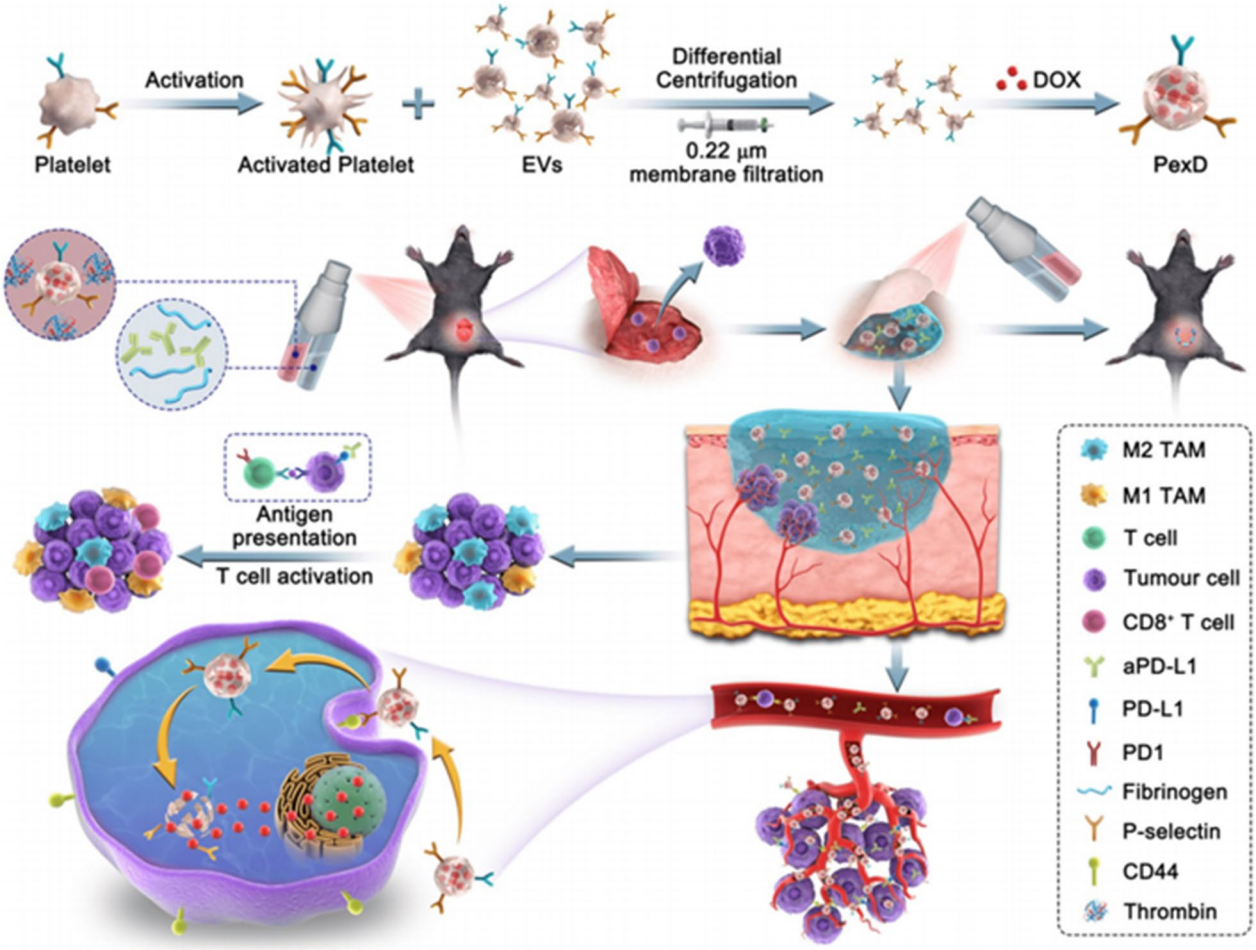
Schematic diagram of dual-chamber nano-hydrogel spray PexD inhibiting post-operative tumor recurrence and metastasis by activating the body's immune system. Reprinted with permission from Ref. [253]. Copyright© 2022, copyright Zhao et al.

Melanoma is a highly malignant tumor mainly found in the skin and the mucous membranes and is mostly treated surgically. However, melanoma has a high recurrence rate due to unavoidable tumor remnants and CTCs (circulating tumor cells) after surgery [249, 250]. Even immunotherapy, including the introduction of PD-L1 blockers, has failed because of low immunogenicity. Immunotherapy combined with chemotherapy is considered a reliable treatment option, but improving its bioavailability and targeting is the key to achieving efficient treatment [251, 252]. Sun et al. [253] proposed a dual-lumen nano hydrogel spray to inhibit postoperative tumor recurrence and metastasis by reviving the body's immune system. Tube A has fibrin and α-PD-L1, and tube B is thrombin and PexD, a platelet-derived extracellular vesicle (Pex) loaded with DOX. The protocol was designed because Pex can capture CTCs as platelets, and the nanosize is more suitable for drug transport. After the tumor excision, the therapeutic drug is sprayed into the incision site in tubes A and B. The thrombin in A and B interacts with fibrin to form a gel in situ at the incision site. This gel functions as a drug release reservoir, releasing high concentrations of PexD and α-PD-L1 into the incision site to ensure their entry into the circulation through the damaged blood vessels on the one hand and promoting wound healing on the other. The released PexD not only induced the tumor ICD and promoted the anti-tumor immune response but also tracked and adhered to CTCs, while α-PD-L1 blocked the PD1/PD-L1 pathway (Fig. 10). Ultimately this gel spray strategy inhibited tumor growth and recurrence and enhanced the survival time of tumor-bearing mice.

The prognosis of the postoperative wound leads to the development of an TIME that induces tumor recurrence and distal metastasis, also after CRC (colorectal cancer) treatment [254, 255]. Chen et al. [256] designed an in situ dual-tube spray gel, iSGels@αOX40, for use after CRC surgery. The A tube consists of PLG-g-mPEG/PBA and the antibody αOX40 that enhances effector t-cell activation and inhibits Tregs. The B tube is TA (tannic acid) with antioxidant, anti-inflammatory and antibacterial activities. After spraying A and B tubes on the tumor site, TA cross-linked with PLG-g-mPEG/PBA to form iSGels with solid adhesion, in which TA could effectively reverse the activity of COX-2 in the tumor immunosuppressive microenvironment. To better represent the therapeutic effect of iSGels@αOX40, the team used subcutaneously and in situ CRC tumor models as experimental subjects. The results showed that iSGels@αOX40 effectively suppressed the expression of COX-2, a key molecule in the tumor immunosuppressive microenvironment, and effectively increased the infiltration of CD4^+^T and CD8^+^T cells at the tumor site. Notably, CD4^+^ and CD8^+^ central memory T cells were significantly elevated in the iSGels@αOX40-treated group, signaling the mobilization of immune memory effects that can prevent tumor recurrence. Although this technique is not based on nanoscale hydrogel spray, it gives a good idea for subsequent CRC immunotherapy.

Since hydrogel nanospray is in its infancy in tumor immunotherapy, there are few relevant literature reports, but this does not prevent it from becoming a hot spot for tumor immunotherapy.

### Hydrogel scaffolds

Although conventional treatment options for cancer are effective in reducing the primary tumor size, they are less effective against metastases or recurrence of the primary tumor. The impediment of TME to conventional treatment has forced researchers to explore it more deeply, and many promising attempts have been made, including tumor vaccines. However, due to the enormous development cost and apparent toxic side effects, it cannot be promoted vigorously in the clinic. Therefore, developing strategies to control primary tumor growth and reverse the TIME is urgent for tumor therapy. Yang et al. [257] designed and constructed a hydrogel scaffold, MRD hydrogel, which could destroy primary tumors and activate the immune response, inspired by bee venom peptides with solid anticancer activity. The MRD hydrogel overcomes the hemolytic effect of bee venom peptide and serves as a good delivery vehicle for the chemotherapeutic drug DOX. TEM showed that MRD hydrogels self-assembled to form a nanofiber network with a diameter of 21.2 ± 4.2 nm. In vitro experiments, clones were almost eliminated in the MRD hydrogel group, and the number of clones was significantly lower than in the other groups. 5.1 and 6.1 times more MRD hydrogels induced cell necrosis than the RD hydrogel and DOX groups and promoted the maturation of DCs well in vitro. In vivo experiments, the team used intratumoral injection to administer the drug to B16-F10 tumor-bearing mice. The results showed that MRD hydrogel had significantly improved anti-tumor effects and significantly prolonged the survival time of the mice compared to the control group and other treatment groups and that such treatment effects were dose-dependent. These effects were attributed to the increased maturation of DCs, activation of NK cells, increased M1/M2 ratio, and increased tumor infiltration in CTL and effector memory T cells in the spleen. While considering the therapeutic effects, the researchers also evaluated the biosafety of the MRD hydrogel, which, as designed, was biocompatible, as evidenced by the fact that blood parameters such as hemoglobin and platelet count, as well as vital organ assessments, remained normal in the treated mice. Ultimately, the MRD hydrogel scaffold not only eliminated the primary tumor but also prevented tumor recurrence and metastasis by generating a memory immune response.

RFA (radiofrequency ablation) is a minimally invasive option that has been approved to treat malignant tumors. It induces ion reciprocal vibration in tumor cells by the high-frequency alternating current to generate heat leading to apoptosis and necrosis [258]. At the same time, studies have also found that the cellular debris produced by the action of RFA can act as tumor-specific antigens to activate the immune system. However, the application of RFA is usually limited, and the risk of tumor recurrence and metastasis remains after treatment [259–261]. This can be attributed to the inadequate antigen presentation by antigen-presenting cells represented by DCs. It has been shown that ROCKs (rho-associated kinases), as key signaling molecules for tumor growth and invasion, are also involved in downregulating the phagocytosis of macrophages and DCs [262]. Chen et al. [263] designed an immunotherapy regimen consisting of PPP (PLGAPEG-PLGA) and ROCKs inhibitor Y27632 based on this regulatory property. The mixture of PPP and Y27632 is a temperature-responsive material that, after injection into the tumor, undergoes aggregation to form a hydrogel scaffold state due to the high temperature generated by the subsequent RFA treatment. Firstly, the large amount of cellular debris produced by the tumor after receiving RFA will attract many antigen-presenting cells. And at this time, the hydrogel acts as a drug reservoir, continuously releasing Y27632 to the outside world, activating the phagocytosis and antigen-presenting ability of dendritic cells and enhancing the immune system response. This process is also accompanied by T-cell infiltration and activation. Notably, PLGAPEG-PLGA has been approved by the FDA for clinical use [264]. Thus, this regimen has a high prospect of clinical translation to expand the limitations of RFA treatment at this stage.

Although hydrogel scaffolds have significant application in cancer vaccines, when considering the cost of preparation, plasticity and storage aspects, Wang et al. [265] used 3D printing technology to prepare hydrogel scaffolds for tumor immunotherapy. This 3D-printed hydrogel scaffold vaccine loaded with immunomodulators has a biomimetic lymphoid structure and can recruit many immune cells. Its immune function was demonstrated in tumor-bearing mice, and the 3D scaffold vaccine recruited many immune cells, including T cells, DCs and macrophages. It also promoted the maturation and antigen-presenting ability of DC cells, which is of great importance for activating the immune response. Although the technology is at the micron level rather than the nanoscale in size, this has not prevented researchers from using it as a starting point to prepare customized nano vaccines with enhanced efficacy that can be manufactured in bulk.

### Hydrogel microneedles

The Transdermal Drug Delivery System (TDDS) has been promoted for clinical use because it avoids first-pass effects, painlessness, convenience, and guaranteed drug activity. This method of transdermal drug delivery for local or systemic treatment has a high status in the biomedical field. However, due to the barrier effect of the skin's stratum corneum, only a few can meet the needs of clinical treatment [266, 267]. Therefore, the concept of microneedle has been proposed to overcome the skin barrier and is widely used in diabetes, wound healing, medical aesthetics and oncology treatment.

**Fig. 11:**
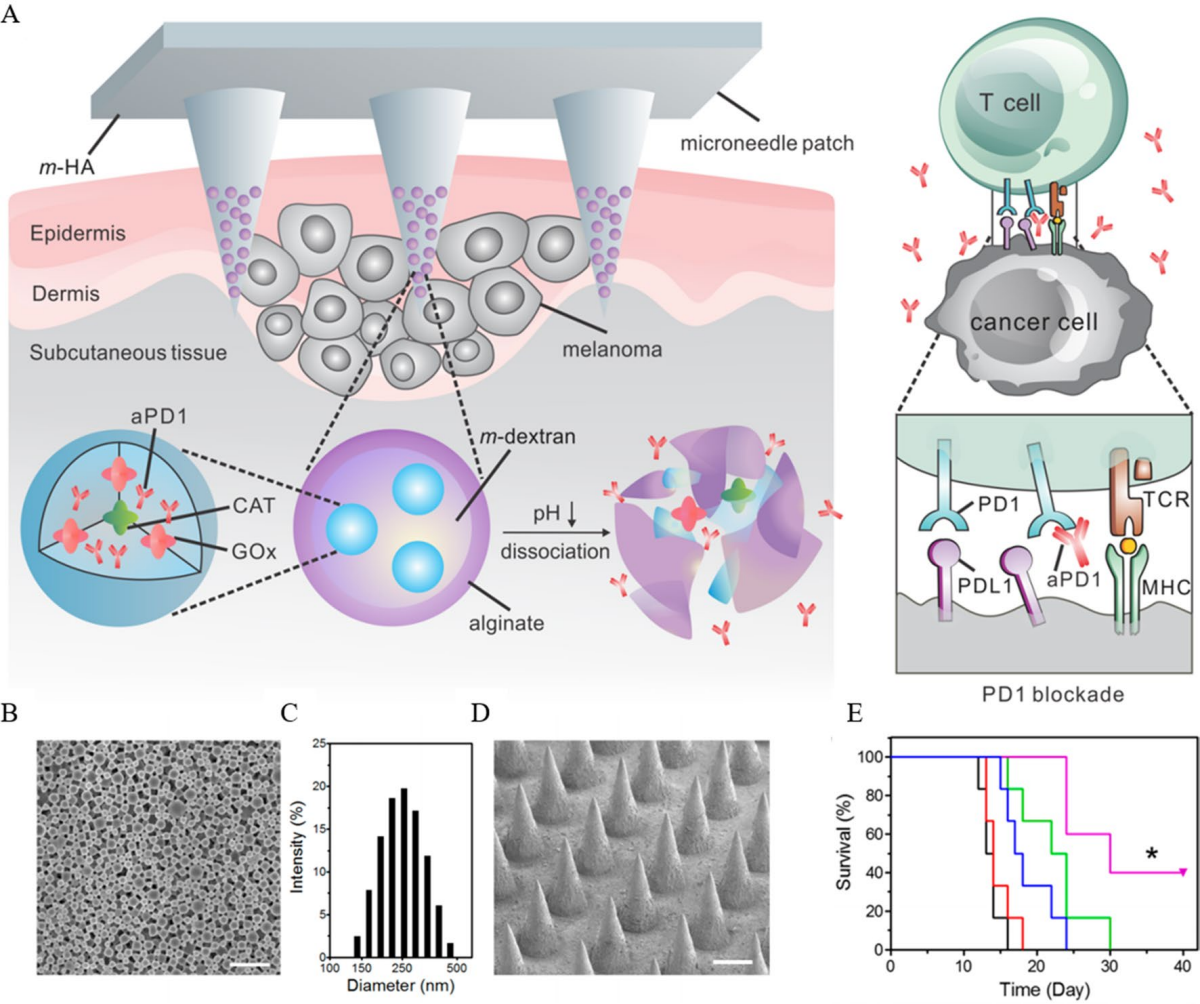
Microneedles MN-GOx-αPD1 for skin cancer immunotherapy. **A** MN-GOx-αPD1 can penetrate the dermis for effective delivery of α-PD1. **B**, **C** SEM images of NPs (Scale bar = 100 nm) and average hydrodynamic dimensions by DLS. **D** SEM images of microneedles (Scale bar = 200 μm). **E** Survival curves of tumor-bearing mice after receiving different treatment strategies (*p < 0.05). Reprinted with permission from Ref. [268]. Copyright© 2016, copyright Wang et al.

The α-PD-1 therapy is an effective treatment option for metastatic melanoma. Still, such results come at the cost of higher doses, which can ultimately lead to high treatment costs and more significant toxicities. Gu et al. [268] developed an MN (microneedle) for melanoma immunotherapy to maximize the drug's efficacy. The MN consists of HA with good biocompatibility, and dextran nanoparticles (NPs) encapsulated with α-PD1 and GOx. When the MN is inserted into the skin, blood glucose reacts with GOx in the NPs with the participation of oxygen to produce gluconic acid with acidity. This acidic environment will also lead to the spontaneous degradation of MN with ph-sensitive properties, resulting in the release of immunoreactive α-PD1 (Fig. 11A). In the characterization assay, the average hydrodynamic size of the NPs was 250 nm. Images showed that each MN was conical with a bottom diameter of 300 um and a height of 600 μm (Fig. 11B–D). In vitro experiments showed that the enzymatic component of the MNs effectively broke down glucose over time to produce gluconic acid and thus lowered pH. In vivo experiments, no biotoxicity was observed even when the concentration of NPs was increased to 1.0 mg mL^−1^, demonstrating its good biocompatibility as a carrier. In the in vivo evaluation of the anti-tumor effect, the MN-GOx-αPD1 group not only exerted the most substantial anti-tumor effect compared to the control and experimental groups but also ensured the survival of 2/5 of the mice within 40 days after treatment (Fig. 11E). In contrast, all mice in the control group died. This promising result was due to a significant increase in CD8^+^ and CD4^+^T cell infiltration in tumors of mice treated with MN-GOx-αPD1. Among them, CD8^+^T cells were three times higher than in free αPD1 treatment, and the expression of CTLA4 was significantly increased in tumor infiltrating lymphocytes. This suggests that this strategy induced more immune effects at the same treatment concentration. With such results, the team added α-CTLA4 to MN-GOx-αPD1. Unexpectedly, tumors were suppressed entirely and remained disease-free for an extended period in about 70% of the mice during the 60-day observation period.

Although PTT allows tumor cells to release TAAs to induce ICD, it also upregulates IDO, an immune-negative regulator, thereby inhibiting the activation of APCs and CD8^+^T activation [269, 270]. With this in mind, Wu et al. [271] designed DMNs (Dissolving microneedles), called CSMNs, that can achieve ICD through PTT and reverse IDO-mediated immunosuppression. The tip of CSMNs is a degradable chitosan NPs (ICG-NPs) encapsulated with ICG, while the bottom is a PVP-PVA gel loaded with 1-MT. The ICG-NPs showed a spherical morphology of about 140 nm under TEM. After being irradiated with 808 nm (0.35 W cm^−2^) NIR for five minutes, the temperature of ICG-NPs increased from 27.3 °C to 54.7 °C, while such a temperature would lead to the disappearance of almost all cells. In vitro experiments revealed that ICG-NPs induced CRT exposure and promoted the maturation of DCs in a concentration-dependent manner. Although a significant upregulation of IDO accompanied this process, it could be well inhibited by loading of 1-MT. In validating efficacy in B16-bearing mice, 1-MT@ICG-NPs-MN + laser induced the highest degree of DCs maturation and the highest concentrations of IFN-γ, TNF-α, IL-12p70 and IL-6 compared to control and other experimental groups. Also, the results showed that PTT enhanced exposure to CRT, which is consistent with previous reports. The final tumors were almost eradicated in the tumor-bearing mice after 1-MT@ICG-NPs-MN + laser treatment, and an 80% survival rate was achieved during the observation period. Despite the enhanced IDO expression by PTT, 1-MT@ICG-NPs-MN + laser induced potent CD8^+^T infiltration and achieved an excellent anti-metastatic effect thanks to the blockade of its downstream pathway by 1-MT.

### Oral hydrogels

It is highlighted that although this section is classified according to the composition of the hydrogel, we have included oral hydrogels in the scope of this review because they are a newly emerging strategy with promising applications in tumor immunotherapy.

**Fig. 12:**
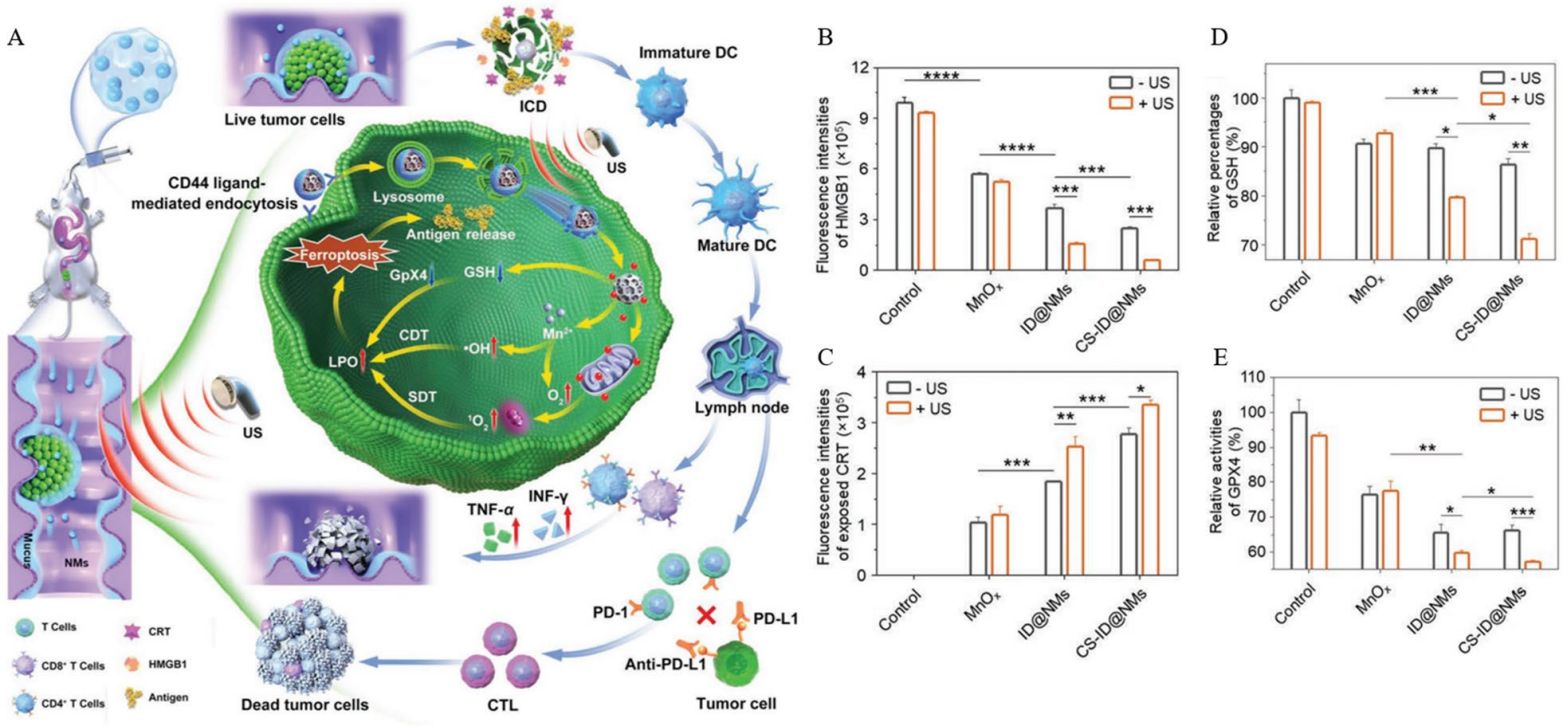
Oral CS-ID@NMs hydrogel has strong anti-tumor effects. **A** Schematic diagram of oral delivery of CS-ID@NMs in the form of oral administration thereby achieving potent tumor immunosuppression. **B**, **C** Quantitative fluorescence intensity of HMGB1 and exposed CRT in each treatment group (scale bar = 100 μm). **D**, **E** The relative amount of intracellular glutathione and the relative activity of GPX4 in each treatment group (*p < 0.05, **p < 0.01, ***p < 0.001, ****p < 0.0001). Reprinted with permission from Ref. [272]. Copyright© 2022, copyright Cao et al.

The highly malignant nature of colon cancer is demonstrated by its robust proliferation, metastasis, and immune escape. Although conventional treatment options such as surgery, chemotherapy and radiotherapy can control the primary, many tumor lesions have limited efficacy in more deeply infiltrated and metastatic tumors. As a common and highly acceptable treatment for colon cancer, oral administration can deliver drugs directly to the lesion but still faces many bottlenecks such as mucus barrier and TIME. To overcome these problems, Xiao et al. [272] proposed the SDT-based oral hydrogel artificial nanomotors (NMs) CS-ID@NMs. This biotherapeutic platform uses mesoporous manganese oxide (MnOx) modified with regenerated silk fibroin (RSF) and CS (chondroitin sulfate) on the surface as a carrier and generated ROS with CDT (chemodynamic therapy) activity by a Fenton-like reaction with H_2_O_2_ highly expressed in TME, and also provides oxygen required for Mn^2+^ to participate in SDT. US-mediated SDT not only has the advantages of non-invasive and high tissue penetration ability but also induces ICD after killing tumor cells, thus well activating the immune response. CS-ID@NMs also contain CSs that are well biocompatible and functionally target CD44, which is highly expressed in colon tumor cell membranes and indocyanine green derivatives (IDs) that have mitochondrial targeting functions. After oral administration, NMs are released from CS-ID@NM/hydrogel in the colon lumen and penetrate the mucus layer into the tumor tissue in the presence of oxygen and US. Subsequently, CD44-mediated endocytosis on the membrane surface of colon cancer cells internalizes NMs. As RSF dissociates in response to pH, ROS, and GSH, IDs rapidly release from NMs and preferentially accumulate around mitochondria. This is immediately followed by the Mn^2+^-mediated killing of tumor cells by CDT and SDT and the generation of tumor debris to activate the immune response (Fig. 12A). In characterization experiments, CS-ID@NMs were measured as spherical particles of approximately 150 nm in size and remained stable when incubated in simulated colonic fluid for 72 h. In vitro assays, CS-ID@NMs exhibited good SDT effects and promoted phagocytosis by tumor cells under the action of US, as envisioned by the investigators.

Meanwhile, Mn^2+^-based CDT had a positive tumor-killing effect, as shown by a 44.7% decrease in cell viability at a 5 μg mL^−l^ concentration of NPs co-incubated with cells for 24 h. The above process was accompanied by decreased HMGB1 content in the nucleus and increased cellular CRT exposure (Fig. 12B, C). Since a decrease in cytoreductive equivalent GSH concentration and an increase in GPX also occurred during this process (Fig. 12D, E), the team found the involvement of ferroptosis in the above process, as a significant increase in LPO content was detected. In vivo experiments, an efficient enrichment of CS-ID@NMs in the intestinal lumen could be observed and, in combination with α-PD-L1, induced more DC maturation and the highest CD8^+^/CD4^+^T cell ratio in the presence of US. At the same time, CS-ID@NMs induced a significant increase in serum concentrations of TNF-α and INF-γ and activation of tumor-infiltrating lymphocytes. These phenomena signify activation of the immune system and ultimately effective suppression of primary and metastatic tumors.

PD-1 was discovered in the late twentieth century and has become the star target of tumor immunotherapy after more than thirty years of development. PD-1/PD-L1 is a hot research topic in ICB, often in the form of antibodies or inhibitors. In recent years, it has been found that peptides have higher tumor tissue penetration compared to the first two forms due to their lower molecular weight, good immunogenicity, specificity and less toxicity. ^D^PPA-1 is a D-peptide that can block PD-1/PD-L1 to some extent, screened by phage display technology, but considering the poor stability, Gao et al. [273] designed a more stable D-peptide OPBP-1 (oral PD-1Binding Peptide 1). In an in vitro assay, OPBP-1 increased IL-2 secretion from CD45^+^Jurkat cells and showed comparable blocking activity to the PD-L1 antibody. Although OPBP-1 did not show an effect on tumor cell growth in the in vitro MTT assay, OPBP-1 demonstrated a potent tumor suppressive effect in the in vivo assay targeting tumor-bearing mice. In further analysis, the researchers found a significant increase in the proportion of CD8^+^T cells in tumors, lymph nodes and spleens after treatment with 0.5 mg kg^−1^ OPBP-1. This regimen resulted in superior tumor suppression by activating the body's immune system due to better bioavailability and plasma half-life.

There is growing evidence that dysbiosis of the gut flora is strongly associated with low response rates to ICB therapy, so some investigators have proposed using probiotics or fecal flora transplants to improve the gut flora in oncology populations. Still, this operation has been questioned because of the risks involved [274–276]. There is evidence that microbial metabolites, including short-chain fatty acids, are involved in the regulation between the intestinal flora and the immune system [277–279]. Moon et al. [280] was the first to suggest through extensive screening that the use of naturally sourced inulin enhances the antitumor effect of α-PD-1. In an in vivo experiment in CT26 colon cancer tumor-bearing mice, inulin gel gavage significantly increased the percentage of CD8^+^T cells and the relative abundance of bacterial genera (e.g., Ackermania spp., Lactobacillus spp. and Roscoe spp) associated with ICD. Meanwhile, the addition of inulin effectively avoided the depletion of Roseburia caused by α-PD-1. Ultimately, oral inulin hydrogel enhanced the antitumor efficacy of α-PD-1, as reflected by the fact that 80% of mice remained tumor-free, significantly higher than the 20% treated with α-PD-1. In conclusion, this dietary fiber-based oral gel strategy provides an excellent pointer to enhancing ICB by integrating intestinal flora with tumor immunity.

## Summary

Different from the traditional anti-tumor protocols such as surgery, radiotherapy and chemotherapy, tumor immunotherapy has been developing rapidly in recent years, with an unstoppable trend waiting at this stage. To a certain extent, they have overcome the incompetence of traditional treatment options in the face of recurrence and metastasis and improved patients' lives with a new attitude. At the same time, it cannot be ignored that tumor immunotherapy is not a panacea. This is attributed to physiological resistance mechanisms such as high expression of CD47 signaling and physical disorders, including abnormal tumor vasculature during tumor evolution. Adding fuel to the fire of tumor immunotherapy is the concerted effort of clinicians and researchers to break the bottleneck of tumor treatment. With such a strong background, nanomaterials have been considered a quality companion for immunotherapy, overcoming a series of difficulties and pushing the attention on immunotherapy to a high level. Since nanomaterials are diverse and involved in tumor immunomodulation in different ways, this review summarizes several currently widely studied nanomaterials, including self-assembled systems, mesopores, biofilm modifications, and hydrogels. It is worth noting that although we intentionally distinguish them, the complex diversity of nanomaterials makes it inevitable that they intersect.

It should not be overlooked that while researchers focus on the functions of tumor immunity-related nanomaterials, they should also pay attention to their long-term toxicity. On the other hand, due to the diversity of nanomaterial compositions, reducing the differences between different synthetic batches of products and ensuring their consistency in terms of morphology, drug loading, and function remains a difficult breakthrough. Although there are numerous basic studies related to these NPs, few of them have been translated into the clinical setting. However, we believe that with the improvement of target screening technology and the synthesis process, nanomaterials will eventually become a strategy to benefit patients.

## Data Availability

The data that support the findings of this study are available from the corresponding author upon reasonable request.

## Abbreviations

APCs: Antigen-presenting cells
CAT: Catalase
CDT: Chemodynamic therapy
CRT: Calreticulin
CTLs: Cytotoxic T-lymphocytes
DCs: Dendritic cells
FDA: Food and Drug Administration
GBM: Glioblastoma
HA: Hyaluronic acid
HIF1-α: Hypoxia-inducible factor 1-α
HMGB1: High mobility group protein 1
HSPs: Heat shock proteins
ICG: Indocyanine green
LA: l-Arginine
MDSCs: Myeloid-derived suppressor cells
MHT: Magnetic heat treatment
MMP-2: Matrix metalloproteinase-2
MN: Microneedle
NO: Nitric oxide
OMVs: Outer membrane vesicles
PA: Photoacoustic
PDT: Photodynamic therapy
PEG: Polyethylene glycol
PET: Positron emission tomography
PTT: Photothermal therapy
RBC: Red blood cells
SDT: Sonodynamic therapy
SIRPα: Signal regulatory protein-α
STING: Stimulator of interferon genes
TAAs: Tumor-associated antigens
TAM: Tumor-associated macrophages
TGI: Tumor growth inhibition
TILs: Tumor infiltrating T lymphocytes
TIME: Tumor immunosuppressive microenvironment
TME: Tumor microenvironment
TiO_2_: Titanium dioxide
TLR-7: Anti-toll-like receptor 7
Treg: Regulatory T cells
US: Ultrasound
ZIF-8: Zeolite imidazolium framework-8
